# Inulin Dehydration to 5‐HMF in Deep Eutectic Solvents Catalyzed by Acidic Ionic Liquids Under Mild Conditions

**DOI:** 10.1002/cssc.202402522

**Published:** 2025-01-24

**Authors:** Salvatore Marullo, Giovanna Raia, Josh J. Bailey, H. Q. Nimal Gunaratne, Francesca D'Anna

**Affiliations:** ^1^ Dipartimento di Scienze Biologiche, Chimiche e Farmaceutiche Università degli Studi di Palermo Viale delle Scienze, Ed. 17 90128 Palermo Italia; ^2^ School of Chemistry and Chemical Engineering Queen's University Belfast Belfast BT9 5AG, Northern Ireland UK

**Keywords:** inulin conversion, 5-HMF, deep eutectic solvents, ionic liquids, biomass

## Abstract

Valorization of carbohydrate‐rich biomass by conversion into industrially relevant products is at the forefront of research in sustainable chemistry. In this work, we studied the inulin conversion into 5‐hydroxymethylfurfural, in deep eutectic solvents, in the presence of acidic task‐specific ionic liquids as catalysts. We employed aliphatic and aromatic ionic liquids as catalysts, and choline chloride‐based deep eutectic solvents bearing glycols or carboxylic acids, as solvents. The reactions were performed in a biphasic system, with acetone as a benign extracting solvent, enabling continuous extraction of 5‐HMF. We aimed to find the best experimental conditions for this transformation, in terms of catalyst loading, solvent, reaction time and temperature to achieve an economical and energy efficient process. We also analyzed the results in terms of solvent viscosity and structural organization as well as catalysts acidity, to elucidate which structural features mostly favour the reaction. Under optimized conditions, we obtained a yield in 5‐HMF of 71 %, at 80 °C in 3 h. Our system can be scaled up and recycled three times with no loss in yield. Finally, comparison with the literature shows that our system achieves a higher yield under milder conditions than most protocols so far reported for the same transformation.

## Graphical abstract

We studied the dehydration of inulin into 5‐HMF in Deep Eutectic Solvents, promoted by task specific ionic liquids, under mild conditions. The reaction occurred in a biphasic system, continuously extracting 5‐HMF. Catalyst and solvents could be reused and we also elucidated which solvent properties are most conducive to the best yields.

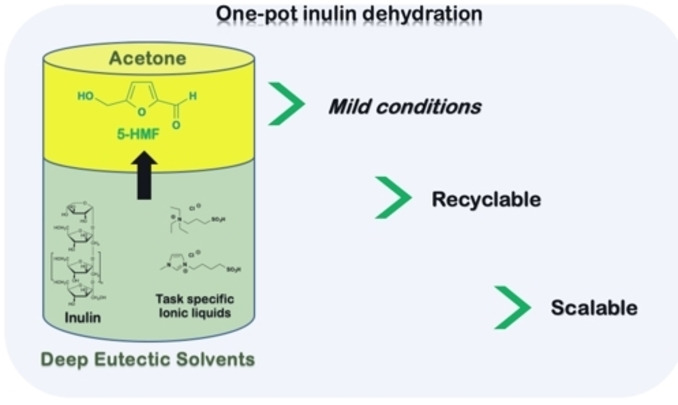

## Introduction

Fossil feedstocks are still the most used sources for both energy and chemicals production, at the base of present‐day standard of living. However, their limited abundance and the problems associated with their large‐scale use, such as global warming and their environmental footprint, have motivated the search for alternative feedstocks that are renewable and with low environmental impact. In this context, in the last years lignocellulosic biomass has recently emerged as the most viable resource for this purpose, due to its inherently low environmental impact, renewability and low cost, particularly when dealing with agricultural waste. Hence, an important focus of this research is aimed at developing effective and sustainable pathways to convert lignocellulosic biomass into products of industrial value. These include biofuels such as bioethanol or biobutanol or chemicals like 2,5‐furandicarboxylic acid or levulinic acid.^[1]^ Such conversions require the presence of catalysts, also including enzymes.^[2]^

For these transformations, it is desirable to use biomass that does not compete with edible crops for soil utilization, such as agricultural waste or non‐grain crops.[^3^, ^4^]

Among the most versatile and sought‐after platform chemicals that are attainable from the conversion of lignocellulosic biomass, a prominent role is played by 5‐hydroxymethylfurfural (5‐HMF).[^5^, ^6^] Readily obtainable from the dehydration of abundant monosaccharides like fructose or glucose, 5‐HMF can be converted into a range of industrially relevant products by different processes like oxidation, reduction, ring opening and also polymerization.[^5^, ^7^]

As aforementioned, there is now a considerable body of work on 5‐HMF production, using a wide range of catalytic systems. These routes mainly consist of dehydration of di‐ and monosaccharides. It is well known that the carbohydrate fraction of lignocellulosic biomass is constituted mostly by polysaccharides with repeating glucose units and their direct conversion into 5‐HMF would be more desirable. However, this is a challenging endeavor, and their subsequent transformation into 5‐HMF often requires harsh conditions, given the recalcitrance of most polysaccharides towards the prior depolymerization to glucose. On the other hand, fructose provides a facile route to 5‐HMF. In this context, using inulin as a substrate for obtaining 5‐HMF can represent a viable solution to this issue. Inulin is a polysaccharide constituted by up to 97 % of fructose units, where the remaining 3 % comprises glucose units. It is characterized by a straight‐chain structure, with an average molecular weight comprised between 3500 and 5500 g/mol. Inulin can be extracted and obtained from several crops, but in the context of the valorization of lignocellulosic biomass, the most important source is a plant known as Jerusalem artichoke.^[8]^ In particular, this plant is the most abundant source of inulin, has a high resistance to weather conditions, and has the ability to grow in soils that are practically useless for the vast majority of edible crops, thus not competing with human nutrition.^[9]^

The conversion of inulin into 5‐HMF is usually carried out under acidic conditions, but can also be accomplished in the presence of enzymes as catalysts.[^10^, ^11^] The acidic catalysts break down the glycosidic bonds, releasing fructose molecules, which, under the same reaction conditions, are readily dehydrated to 5‐HMF. Consequently, inulin has the advantage over other polysaccharides of releasing practically the most reactive monosaccharide.

Despite these advantageous features, to date the utilization of inulin as a feedstock for this task has been largely underexplored. Most of the works reported in the literature employ dimethyl sulfoxide (DMSO) or its mixtures as solvents, with a variety of Brønsted or Lewis acid catalysts, including task specific ionic liquids (TSILs). These are ionic liquids functionalized with a catalytically active moiety, typically a sulfonic acid group in this case. From the standpoint of green chemistry, the solvent plays a pivotal role in determining the sustainability of the process, and in this context, we have studied and evaluated the beneficial effect of non‐conventional solvents for the dehydration of sugars into 5‐HMF, such as ionic liquids[^8^, ^12^, ^13^] and deep eutectic solvents (DES).^[14]^

These kinds of solvents[^15^, ^16^, ^17^, ^18^] are particularly suitable for carbohydrate conversions, as they provide high solubilizing power for carbohydrates and better dispersion of polysaccharides, inertness of solvent system to reaction conditions and, in principle, they can be recycled.

Compared with other carbohydrate‐rich feedstocks, there is a significant gap in the use of such non‐conventional solvents for the conversion of inulin, particularly for DES.^[19]^ DES are solvents constituted by mixtures of components, with a definite melting point, which is significantly lower than the one predicted by the models for ideal mixtures.^[20]^ They are typically constituted by simple and cheap species as components, often of natural origin, and possess beneficial properties such as low vapor pressure and low flammability. More specifically, DES are generally constituted by a hydrogen bond donor (HBD) and a hydrogen bond acceptor (HBA) component, which can confer to them a distinct nanostructural organization underpinned by hydrogen bond networks.^[21]^

**Figure 1:**
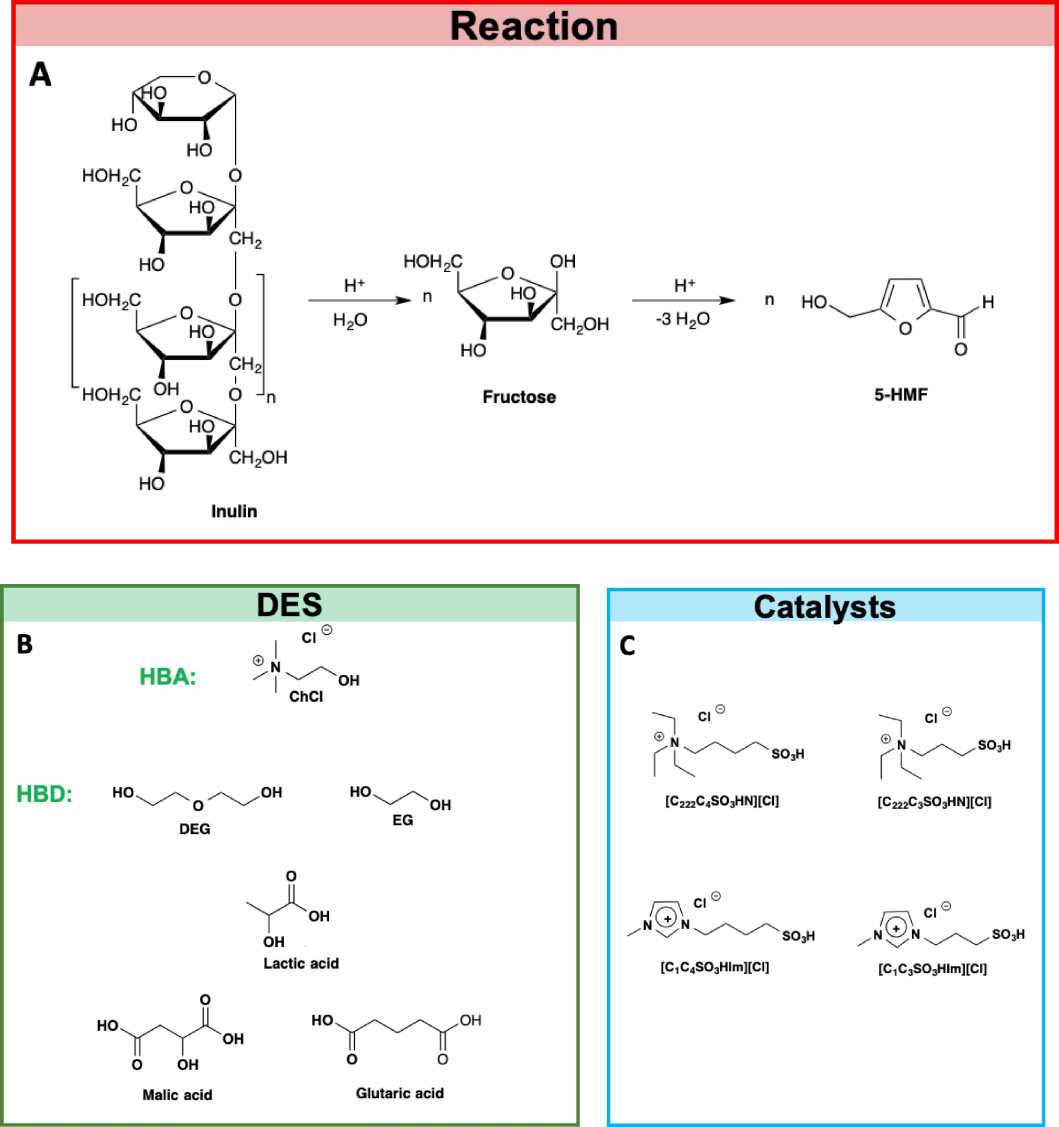
a) Reaction pathway, b) structures of solvents and c) catalysts.

Based on these considerations, we studied the transformation of inulin into 5‐HMF, in the presence of sulfonic acid functionalized TSILs as catalysts, in solution of cholinium chloride‐based DES, bearing both glycols or carboxylic acids as the HBD (Figure 1).

In particular, glycol‐based DES were characterized by a different number of oxygen atoms and methylene units in the HBD, which in turn can affect their structural organization.^[22]^ Moreover, for the other DES, both mono‐and dicarboxylic acid were considered, to evaluate the impact of the presence of a second acidic moiety. Finally, all the catalysts were TSILs functionalized with a sulfonic acid moiety, differing for the aromatic or aliphatic nature of the cation, as well as the number of carbon atoms interposed between the cationic head and sulfonic acid group.

The different nature of the cationic head could have significant repercussions on the toxicity of the solvent medium, considering the lower toxicity of aliphatic ILs compared with the corresponding aromatic ones.^[23]^ On the other hand, the spacer length, could influence cation conformational flexibility and, consequently, its performance.

The catalysts chosen were used due to their ability to provide acidic sites which are less corrosive than mineral acids. Moreover, as clearly shown in literature, despite being less acidic than H_2_SO_4_, sulfonic acid‐functionalized ILs promote the hydrolysis of inulin more efficiently, due to the synergistic action of the SO_3_H‐moiety and IL anions in breaking the glycosidic bond between fructose units.^[24]^

The reactions were performed in a biphasic system employing acetone as the extracting solvent. This allowed the continuous extraction of the product, significantly reducing its degradation. We optimized the reaction conditions, in terms of catalyst loading, temperature and reaction time, for each catalyst.

We also carried out viscosity and resonance light scattering investigation on the DES and their solutions with the catalysts, to elucidate the effects of viscosity and solvent structural organization on the yield in 5‐HMF. In addition, we assessed the influence exerted by the acidity of the catalysts, as expressed by their Hammett acidity function, *H*_0_. In general, we obtained high yields in 5‐HMF, carrying out the reaction at the relatively low temperature of 80 °C, with the highest yield amounting to 71 %, observed in ChCl : EG, in the presence of [C_1_C_4_SO_3_HIm][Cl]. Finally, we successfully carried out the scale‐up of the reaction, while the catalyst and solvent could be reused up to three times without loss in yield. Comparison with the available literature, shows that our protocol is competitive with most of systems reported for this transformation in terms of yields, mild conditions and reaction time. Finally, in this work we coupled the optimization of the reaction with the elucidation of the properties of the solvents most conducive to the best reaction outcome.

## Experimental Section

### Materials

Commercially available cholinium chloride, ethylene glycol, diethylene glycol, glutaric acid, (±) malic acid, (±) lactic acid, inulin (from chicory root, average MW 5000 g/mol), triethylamine, 1,4‐butanesultone, 1,3‐propanesultone, 1‐methylimidazole, and hydrochloric acid were used without further purification. The solvents methanol and acetonitrile were purchased and used without further purification. Deep eutectic solvents were prepared as previously reported.[^14^, ^25^] Stoichiometry of the ChCl : HBD DES was 1 : 2 for the glycol‐based DES and 1 : 1 for the carboxylic acid‐based ones.

The catalyst [C_1_C_4_SO_3_HIm][Cl] was prepared by following a reported procedure.^[13]^ The other catalysts were prepared by a modification of the reported procedure.^[13]^

### General Procedure for the Synthesis of Catalysts

1 g of 1,4‐butanesultone or 1,3‐propanesultone was dissolved in 5 mL of acetonitrile and the resulting solution was added to the suitable amine (1.2 eq.), dissolved in 5 mL of acetonitrile. The resulting mixture was then heated at 80 °C overnight, under stirring. Subsequently, the reaction mixture was allowed to cool down to room temperature and the solvent was evaporated at reduced pressure. The residue was washed with portions of diethyl ether (3 × 5 mL), and evaporation of the remaining solvents yielded the relevant zwitterion as a colorless solid. The solid obtained was subsequently dissolved in 5 mL of methanol, the stoichiometric amount of HCl was added, and the resulting solution was stirred at room temperature overnight. Evaporation of the solvent, followed by washing of the residue with diethyl ether (3 × 5 mL), afforded the catalysts as colorless solids or pale‐yellow oils.

#### Triethyl‐(1‐Sulfobutyl)Ammonium Chloride [C_222_C_4_SO_3_HN][Cl]

Yield: 98 %. Pale yellow oil. ^1^H NMR: (400 MHz, D_2_O) δ=1.11 (9H, t, *J*=8 Hz), 1.66 (4H, m), 2.81 (2H, m), 3.06 (2H, m), 3.13 (6H, m) ppm. ^13^C NMR: (400 MHz, D_2_O) δ=6.6, 19.9, 21.2, 49.9, 52.6, 55.2 ppm.

#### Triethyl‐(1‐Sulfopropyl)Ammonium Chloride [C_222_C_3_SO_3_HN][Cl]

Yield: 97 %. Colorless solid. m. p.: 65–67 °C. ^1^H NMR: (400 MHz, D_2_O) δ=1.13 (9H, t, *J*=8 Hz), 1.98 (2H, m), 2.83 (2H, t, *J*=8 Hz), 3.17 (8H, m) ppm. ^13^C NMR: (400 MHz, D_2_O) δ=6.6, 18.8, 47.2, 52.7, 54.7 ppm.

#### 1‐methyl‐3‐(1‐Sulfopropyl)Imidazolium Chloride [C_1_C_3_SO_3_HIm][Cl]

Yield: 99 %. Colorless oil. ^1^H NMR: (400 MHz, D_2_O) δ=2.19 (2H, m), 2.80 (2H, m), 3.77 (3H, s), 4.24 (2H, t, *J*=8 Hz), 7.33 (1H, m), 7.40 (1H, m), 8.84 (1H, s) ppm. ^13^C NMR: (400 MHz, DMSO) δ=26.4, 36.2, 47.3, 48.2, 122.8, 124.1, 137.2 ppm.

### TGA Measurements

The temperatures of decomposition, as well as the mass loss, were measured using a TGA Q5000 thermogravimetric analyzer (TA instruments), at a heating rate of 5 °C min^−1^ under nitrogen flow. The maximum values of the DTGA curves of each thermogram were used as a measure of the decomposition temperature. The mass loss was calculated from the area of the peaks exhibited by the DTG curves.

### RLS Measurements

RLS measurements were carried out with a spectrofluorometer employing a synchronous scanning mode in which the emission and excitation monochromators were preset to identical wavelengths. The RLS spectrum was recorded from 300 to 700 nm, with both excitation and emission slit widths set at 3.0 nm. In the case of ChCl : MA solutions, excitation and emission slit widths were set at 1.5 nm.

### Viscosity Measurements

Viscosity of the DES and DES‐catalyst mixtures were measured on a rheometer, employing a plate‐plate geometry apparatus in the temperature range comprised between 15 and 80 °C.

### Conversion of Inulin into 5‐HMF

A suitable amount of inulin was weighed in a vial equipped with a hermetic lid, and then 150 mg of solvent was added. In another vial, a suitable amount of catalyst was added with 200 mg of solvent. Both mixtures were heated at 80 °C for 20 min, under stirring, to ensure complete dissolution. Subsequently, the appropriate amount of catalyst solution was added to the inulin solution, together with 1 mL of acetone. Then, the reaction mixture was kept at 80 °C under stirring, for an appropriate reaction time. Subsequently, the reacting mixture was allowed to cool down at room temperature, and the acetone phase appeared well separated from the DES phase. The supernatant was withdrawn, and diluted in methanol, recording the UV‐vis spectrum, to determine the amount of 5‐HMF based on a calibration curve previously obtained. These spectra were recorded using a methanol/acetone mixture at the same composition present in the samples, as a blank. To verify if any residual 5‐HMF was present in the DES phase, a weighed aliquot of this latter was dissolved in 5 mL of methanol, and the presence of 5‐HMF was assessed as described above. We were not able to determine the conversion value, because the DES caused clogging of the HPLC injection system.

To isolate 5‐HMF, the acetone phase was evaporated and the residue was re‐dissolved in 3 mL of ethyl acetate. This solution was percolated through a pad of silica (3 cm), subsequently removing the solvent by evaporation.

## Results and Discussion

### Synthesis of the Catalysts

**Figure 2:**
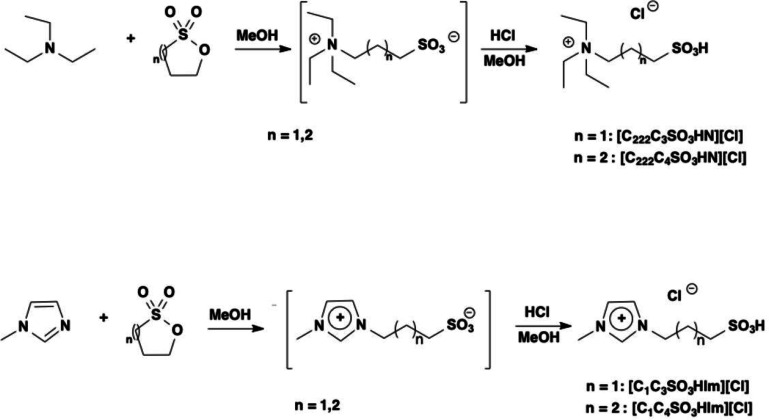
Synthesis of the TSILs used as catalysts.

The TSILs used as catalysts were prepared following a reported two‐step procedure, outlined in Figure 2.^[13]^

In the first step, the appropriate amine, 1‐methylimidazole or triethylamine, was reacted with 1,4‐butanesultone or 1,3‐propanesultone, to obtain the relevant zwitterion. This latter was further treated with the stoichiometric amount of HCl, to introduce the sulfonic acid moiety.

To further characterize the catalysts, and to ensure that they can endure the reaction conditions, we evaluated their thermal stability by thermal gravimetric analysis (TGA). The relevant plots are reported in Figure S1 while the decomposition temperatures are reported in Table 1.

**Table 1:** Decomposition temperatures obtained from TGA measurements.

TSIL	Decomposition temperature (°C)
[C_222_C_3_SO_3_HN][Cl]	164
[C_222_C_4_SO_3_HN][Cl]	179
[C_1_C_3_SO_3_HIm][Cl]	215
[C_1_C_4_SO_3_HIm][Cl]	163

Results reported in Table 1 show that the catalysts used are suitably thermally stable with decomposition temperatures between 163 °C and 215 °C, which are considerably higher than the temperature employed for the conversion of inulin to 5‐HMF (see later).

### Conversion of Inulin into 5‐HMF: The Solvent Effect

To conduct a rapid screening of the catalytic activity of our systems, we firstly carried out preliminary kinetic experiments, determining the yield in 5‐HMF at selected times, from 15 min to 180 min, under the following reaction conditions: 20 mg of inulin, 250 mg of DES, n_cat_/n_inu_=1/3, T=80 °C.

**Figure 3:**
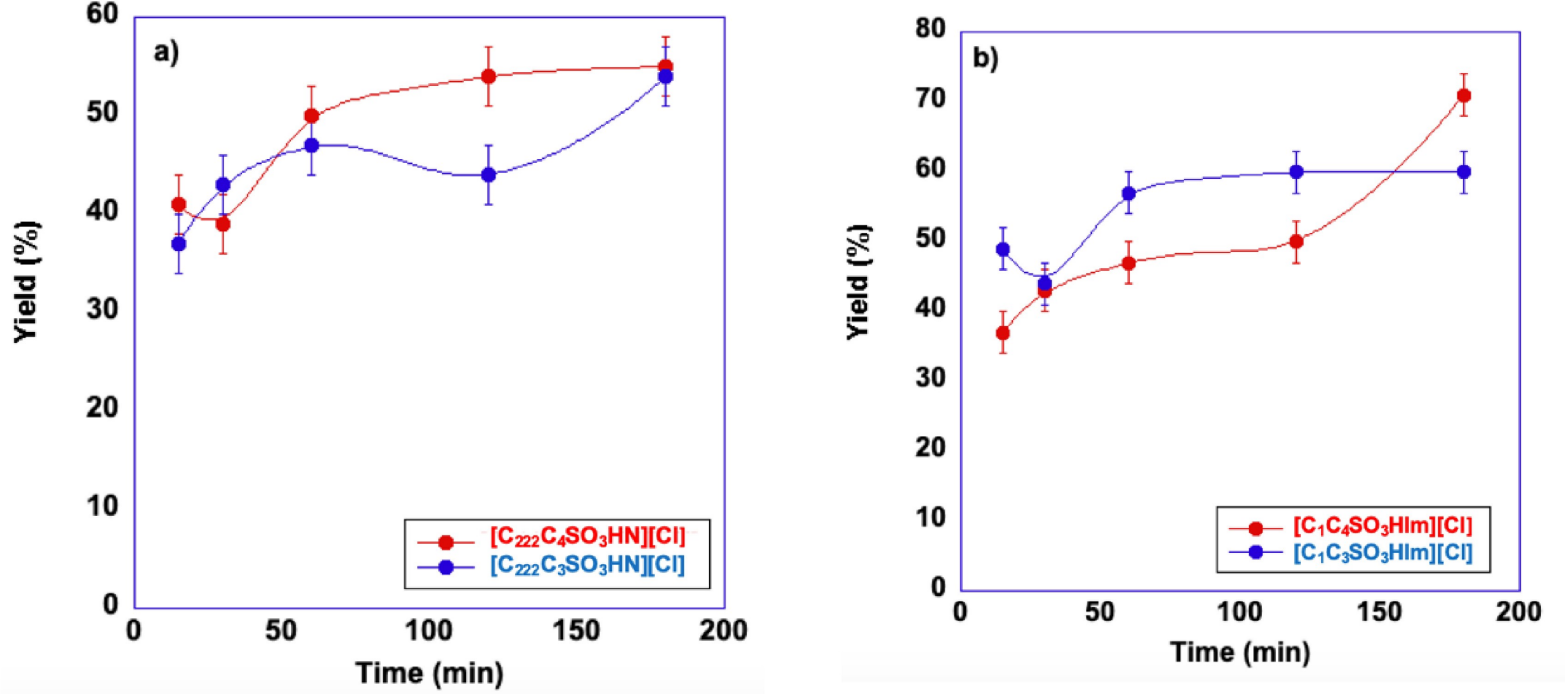
Plots of the yields in 5‐HMF observed at 80 °C in ChCl : EG in the presence of a) ammonium‐based TSILs and b) imidazolium‐based TSILs. Yield values are reproducible within ±4 %. Lines are drawn as a mere visual guide.

As a representative case, we report in Figure 3 the results observed in the DES ChCl : EG, while the other ones are reported in Figure S2 and Tables S1–S5.

Perusal of the plots reported in Figure 3 and S2 shows that, regardless of the catalyst and solvent used, the yields in 5‐HMF approach 40 % only after 15 min of reaction, pointing out a quite fast hydrolysis step from inulin to fructose. Such observation is consistent with literature reports concerning the same reaction conducted in ILs.^[26]^ In particular, the minimal but unavoidable amount of residual water in such solvents is sufficient to initiate the hydrolysis process, which is then further accelerated by the water formed in the dehydration step of fructose.^[26]^ To support this hypothesis, we recorded ^1^H NMR spectra of the reaction mixtures in ChCl : EG, in the presence of all catalysts after 2 min, (Figure S5). These spectra already show the signals from 5‐HMF, again suggesting the fast hydrolysis of the polymer and the subsequent dehydration of fructose. On the other hand, ^1^H NMR spectra of the mixture at the end of the reaction, (3 h, Figure S5), show the prominent presence of 5‐HMF signals. In both cases, it is not possible to distinguish the signals of the inulin proton signals, because they are covered by the ones of the DES, present in large excess.

**Figure 4:**
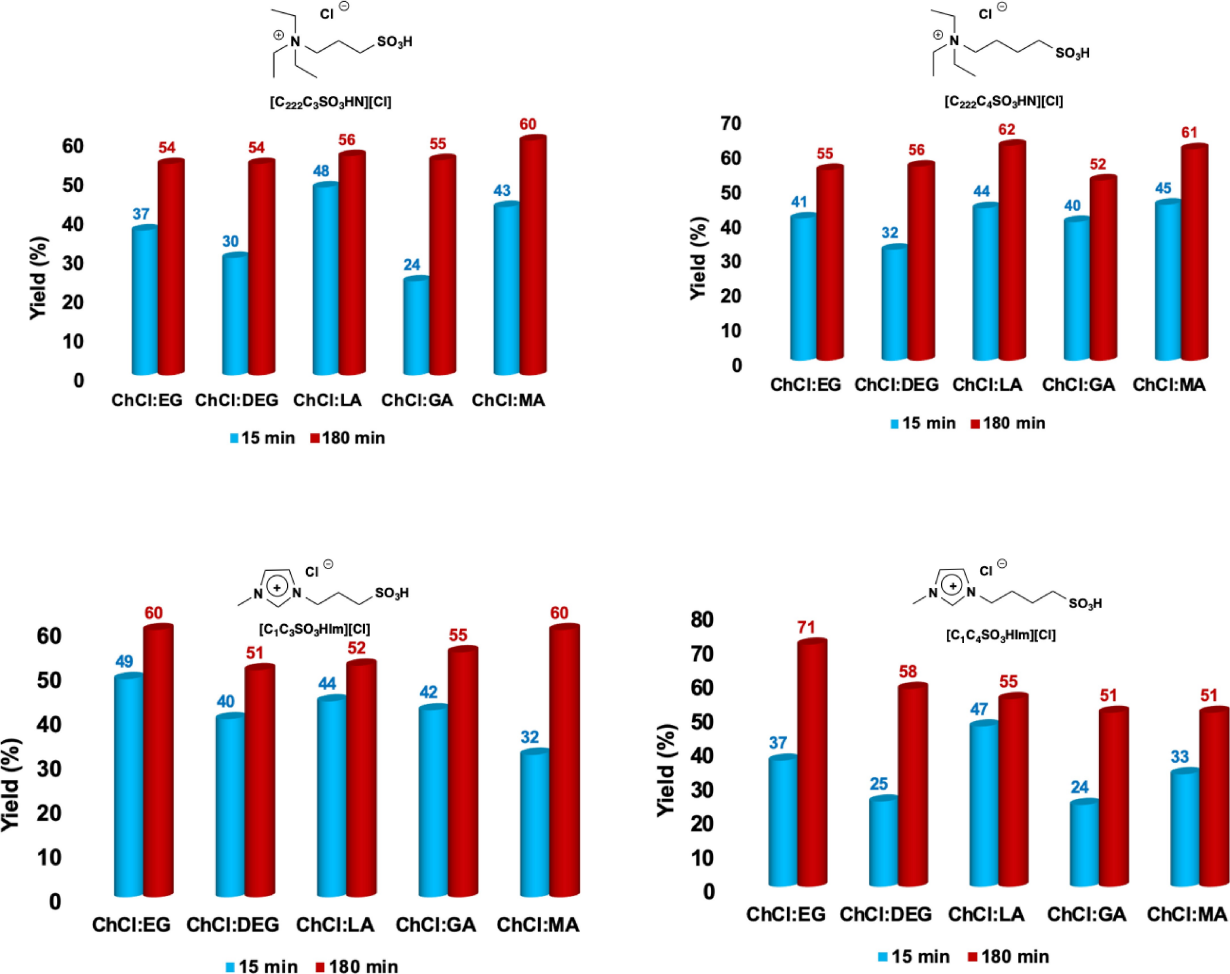
Plots of the yields in 5‐HMF observed at 80 °C in DES after 15 min and 180 min. Yield values are reproducible within ±4 %.

The highest yield, 71 %, was achieved in ChCl : EG, after 3 h, in the presence of [C_1_C_4_SO_3_HIm][Cl], as the catalyst. Moreover, with the sole exception of the reactions conducted in ChCl : DEG, in the other cases the yield gradually increased, reaching a maximum value of 55 %. Finally, in ChCl : DEG, these preliminary tests, indicated that the yield did not exceed 50 %. To better highlight the difference detected in terms of the reaction kinetics, in Figure 4 we report the yields obtained after 15 min and 180 min, in the presence of all the catalysts used. In particular, the value obtained after 15 min is related to the initial rate of the process, while the value found at 180 min is related to the final yield value.

Obviously, before any consideration, it is important to remember that the acid‐containing DES might promote the reaction in their own right. Consequently, to evaluate this, we conducted blank tests, in which inulin was allowed to react in solution of ChCl : LA, ChCl : MA and ChCl : GA, for 3 h, under the same reaction conditions already described, obtaining yields in 5‐HMF equal to 40 %, 35 % and 30 %, respectively. In all cases, the yield obtained is lower than the one observed in the presence of any of the catalysts used in this work, highlighting the need of the presence Brønsted acidic sites for the one‐pot conversion of inulin to 5‐HMF.^[8]^

With the above premises in mind, examination of the results reported in Figure 4, allows us to draw some comments. First of all, in the presence of catalysts like [C_222_C_3_SO_3_HN][Cl], [C_222_C_4_SO_3_HN][Cl] and [C_1_C_3_SO_3_HIm][Cl], the yields value appear almost independent of the solvent used, whereas, in the presence of [C_1_C_4_SO_3_HIm][Cl], more significant variations in yield are detected as a function of the solvent used. The highest yield in 5‐HMF is found in solution of a DES devoid of acid, as ChCl : EG, whereas the lowest ones are collected in ChCl : GA and ChCl : MA.[^15^, ^27^] The lower yields obtained in the latter cases could be due to the acidity of the solvent medium. Indeed, acidic DES are both able to promote the formation of 5‐HMF, and also catalyse its decomposition. The trend observed could be the result of the balance of the above contrasting processes.

In general, with the only exception of the reactions carried out in the presence of [C_1_C_4_SO_3_HIm][Cl], the yield in 5‐HMF were ~40 % after 15 min, and to ~50 % after 3 hours. Given that with this catalyst, more pronounced differences as a function of the solvent were detected, it is possible to make some comments. First, as already said, we will consider the yields in 5‐HMF after 15 min, which are related to the initial rate of the process. In particular, the values increase in the order ChCl : GA=ChCl : DEG<ChCl : MA≈ChCl : EG<ChCl : LA. We can explain this sequence by correlating it to solvent‐related properties. In particular, it derives from the concomitant action of contrasting factors like the presence of acidic centers in the solvent (favourable) and solvent viscosity (unfavourable). It is worth noting that, although the acidic DES are composed by weak acids, with aqueous p*K*a′s comprised between 3.40 and 3.86,^[27]^ in DES solution their acidity is sufficient to promote the reaction to a certain extent, without added catalyst (see above). On the other hand, using the viscosities of the neat DES measured at 298 K as an indicative guide, we can see that this balance mostly affects the acid‐bearing DES. Indeed, the reaction is initially slower in ChCl : GA, despite providing the highest number of acidic sites, because of its highest viscosity, amounting to 2015 mPa s.^[28]^ Accordingly, the initial yields detected in solution of the DES ChCl : MA and ChCl : LA, parallel their decrease in viscosity (η=1389 mPa⋅s, 262 mPa⋅s for ChCl : MA^[28]^ and ChCl : LA,^[29]^ respectively). Similarly, the same trend is followed by the glycol‐based DES, with the yield in 5‐HMF found in ChCl : EG higher than that detected in ChCl : DEG, according to the higher viscosity of the latter (η=31 mPa⋅s and 55 mPa⋅s for ChCl : EG^[30]^ and ChCl : DEG,^[31]^ respectively). To further confirm this observation, we measured the viscosities of the DES at the same temperature used for the reaction. The plots relevant to these measurements are reported in Figure S3. The trend of viscosity of the DES at 80 °C closely parallels the one for the values obtained at 25 °C (ChCl : EG=ChCl : DEG<ChCl : LA<ChCl : GA<ChCl : MA; η=8.6 mPa s for ChCl : EG and ChCl : DEG, 68.6 mPa s for ChCl : LA, 500.8 mPa s for ChCl : MA and 107.1 mPa s for ChCl : GA, respectively), with viscosity differences of smaller magnitude, supporting the hypothesis that experimental trend is the result of the balance of the two contrasting factors mentioned above.

Regarding the yields in 5‐HMF obtained after 180 min, we can observe rather similar values for the acid‐bearing DES, and higher values for the glycol‐based ones. These results clearly reflect the capacity of the former to promote side processes like the acid‐catalysed degradation of part of the 5‐HMF formed. On the other hand, ChCl : EG, and ChCl : DEG do not possess further acidic sites, and therefore this issue is more limited in these solvents, giving the best yield in 5‐HMF, 71 %, in solution of ChCl : EG, in the presence of [C_1_C_4_SO_3_HIm][Cl] as a catalyst.

### Optimization of the Experimental Conditions

**Figure 5:**
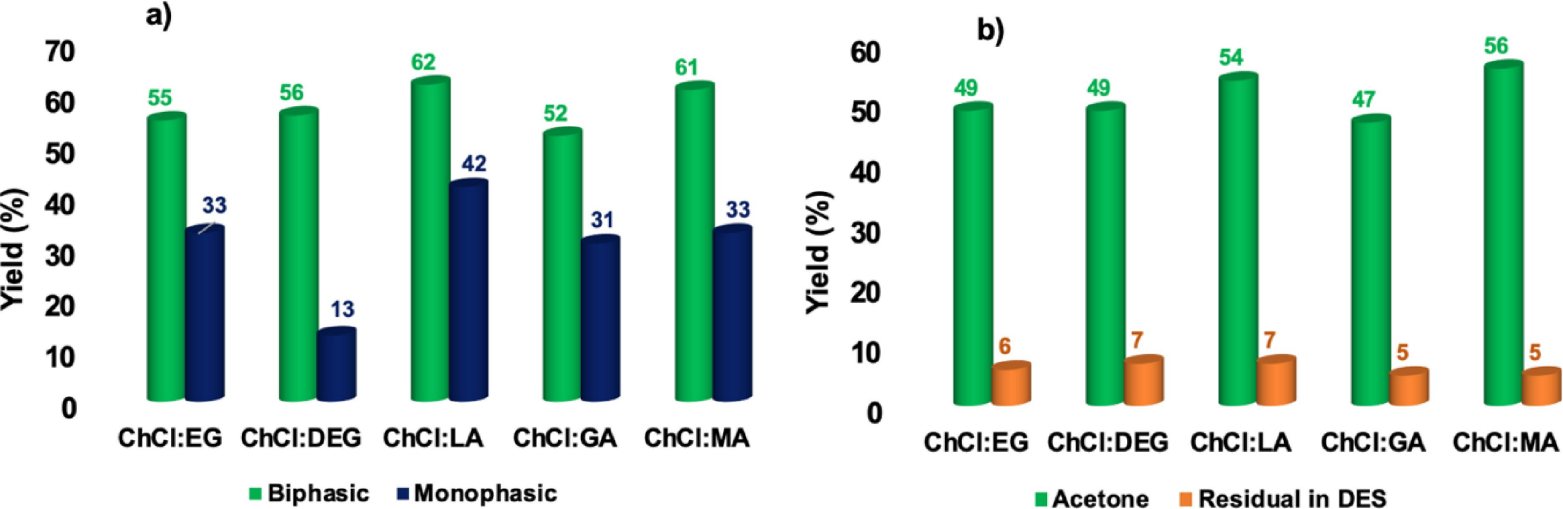
Plots of the yield in 5‐HMF obtained at 80 °C, 3 h, in the presence of [C_222_C_4_SO_3_HN][Cl], as catalyst a) in mono‐ and biphasic systems DES‐acetone and b) in biphasic systems as extracted in acetone and residual in DES phase.

Having gathered preliminary information of the effect of catalysts and solvents on the reaction, we proceeded to further optimize the experimental parameters. Firstly, we wanted to confirm that acetone is a suitable extracting solvent for this reaction. To this aim, we carried out the reaction also in a monophasic system, and compared the results obtained with the ones obtained in the biphasic system presented above. As a representative case, we report in Figure 5a the results obtained in the mono‐ and biphasic reaction mixtures in the presence of [C_222_C_4_SO_3_HN][Cl], after 3 h, under the same experimental conditions described above.

The plots in Figure 5a clearly shows that, irrespective of the solvent, the yield in 5‐HMF detected in the biphasic system is always significantly higher than the ones found in the monophasic system, indicating that the presence of acetone favours the reaction by removing it from the reacting mixture. The extraction of 5‐HMF to acetone phase avoids its prolonged contact with the acidic reaction mixture, and consequently limits the degradation pathway significantly. In this vein, we also wanted to verify if 1 mL of acetone was able to extract all of the 5‐HMF formed, and that no further extraction step is required. To this aim, for all the reactions conducted in biphasic system, we checked for the residual presence of 5‐HMF in the DES phase. As can be seen in the plot in Figure 5b, the residual amount left in the DES phase is rather negligible, confirming the suitability of acetone as an extracting solvent.

To further evaluate the purity of the 5‐HMF obtained from the biphasic system, we evaporated the acetone phase at the end of the reaction, dissolving the residue in 3 mL of ethyl acetate. We percolated this solution onto a small pad of silica and recorded the ^1^H NMR spectra of the residue. The spectrum obtained, reported in Figure S6, shows the only the signals ascribable to 5‐HMF.

### Effect of the Cation Head of the Catalyst

**Figure 6:**
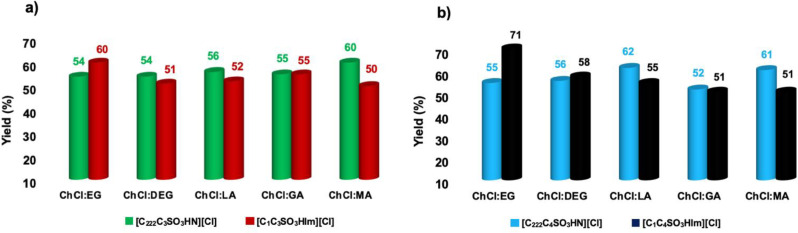
Comparison of the yields in 5‐HMF obtained at 80 °C, 3 h, in the presence of a) [C_222_C_3_SO_3_HN][Cl], and [C_1_C_3_SO_3_HIm][Cl] and b) [C_222_C_4_SO_3_HN][Cl], and [C_1_C_4_SO_3_HIm][Cl] as catalysts.

To draw a correlation between the structure of the catalyst and the yields obtained, we turn our attention to the effect of the structure of the cationic head of the catalyst, comparing the results obtained in the presence of aliphatic or aromatic TSILs. In this case, we consider the value of yields obtained after 3 h (Figure 6). In Figure 6a and 6b, we report separately the yields obtained in the presence of TSILs sharing the same spacer length, *i. e*. 3 and 4 carbon atoms respectively.

Notably, in both cases, a similar trend can be observed when the catalyst cation changes from aliphatic to aromatic. More specifically, the aliphatic TSILs give the highest yield in ChCl : MA, whereas the aromatic ones achieve the highest values in ChCl : EG. In solution of the other DES, the differences are less pronounced, and in ChCl : GA, practically no difference is detected as a function of the nature of the cation head of the catalyst.

### Effect of the Alkyl Spacer Length

Next, we used the same approach to discuss the effect exerted by the length of the spacer chain between the cation head and the sulfonic acid moiety. The relevant plots of the yields are reported in Figure S4a for the imidazolium‐based TSILs and in Figure S4b for the ammonium‐based ones. Looking at these plots clearly shows that the effect of the different spacer length is negligible in most cases, the only exception being that of the imidazolium TSILs in solution of the diol‐based DES ChCl : EG and ChCl : DEG. In such solvents, a longer spacer appears to consistently lead to higher yields. In particular, the yields in 5‐HMF go from 60 % to 71 % in ChCl : EG, as the spacer length is changed from 3 to 4 carbon atoms. In ChCl : DEG, the same structural variation is associated with an increase in yield from 51 to 58 %. Thus, the effect of the alkyl spacer length is rather marginal, with the exception of the aromatic TSILs in ChCl : EG. This suggests a certain importance of van der Waals interactions. It is indeed reported that van der Waals interactions are important in underpinning the structural organization of both ionic liquids^[32]^ and DES.^[33]^ This effect is more visible in the presence of the aromatic IL catalyst, due to their higher degree of organization compared with their aliphatic counterparts.

### Effect of the Catalyst Acidity

It can be interesting, at this point, to evaluate the acidity of the catalyst to verify if there is a correlation with the yields obtained. We therefore evaluated the Hammett acidities of the catalysts in methanol, using methyl orange as spectroscopic probe. We are aware that the values detected in methanol can be different from those in DES, but we chose to use the acidities in methanol as an indicative guide, because the p*K*_a_ of methyl orange in DES is not known. Although the behavior of acids and bases could be different in non‐conventional solvents,[^34^, ^35^] this approach has nonetheless provided valuable information on different kinds of reactions in non‐conventional solvents, both acid‐ and base catalysed.[^36^, ^37^, ^38^] The values of the Hammett acidities obtained are reported in Table 2.

**Table 2:** Hammett acidities of the catalysts used.

Catalyst	*H*_0_
[C_222_C_3_SO_3_HN][Cl]	3.24
[C_222_C_4_SO_3_HN][Cl]	3.83
[C_1_C_3_SO_3_HIm][Cl]	3.42
[C_1_C_4_SO_3_HIm][Cl]	3.63

The values reported in Table 2 clearly show that acidity of the catalysts changes within a narrow range. In general, the lengthening of the alkyl spacer induces a corresponding increase in the *H*_0_ values. However, with the exception of the aromatic catalysts in ChCl : EG and ChCl : DEG, there is no obvious correlation between the acidity of the catalyst and 5‐HMF yield. This absence of correlation agrees with similar observations regarding the conversion to 5‐HMF from monosaccharides^[13]^ and also inulin.^[24]^

In summary, the whole of the findings discussed above, point to a rather articulated trend of yields as a function of the solvent and catalyst used. The most prominent correlation found between initial reaction rate and solvent properties involves solvent viscosity. On the other hand, the most pronounced influence of the catalyst structure on the yield, is exerted by the alkyl spacer length of aromatic IL catalysts.

In addition, we have shown that the efficiency of the catalysts considered, to promote the conversion of inulin into 5‐HMF, is heavily dependent on the solvent used. For this reason, to summarize all the previous findings, we report in Table 3, the experimental conditions leading to the highest yield in 5‐HMF achieved in each DES.

**Table 3:** Reaction times and catalysts leading to the highest yields in 5‐HMF, for each DES at 80 °C.

DES	Time (min)	Catalyst	Yield in 5‐HMF (%)
ChCl : EG	180	[C_1_C_4_SO_3_HIm][Cl]	71
ChCl : DEG	180	[C_1_C_4_SO_3_HIm][Cl]	58
ChCl : LA	120	[C_222_C_4_SO_3_HN][Cl]	52
ChCl : MA	120	[C_222_C_4_SO_3_HN][Cl]	52
ChCl : GA	120	[C_222_C_4_SO_3_HN][Cl]	47

With these results at hand, we wanted to investigate if they could reflect differences in structural organization of the solvents. It is indeed widely known that DES are generally characterized by a distinct microstructure.[^21^, ^39^, ^40^] To this aim, we carried out Resonance Light Scattering measurements (RLS). This is a scattering technique which can be used to investigate aggregates in solution, given that the I_RLS_ is correlated to the size of the aggregates, with higher intensities suggesting larger aggregates.[^41^, ^42^]

Consequently, RLS has been employed in studying a diverse range of aggregated systems,[^43^, ^44^, ^45^] and we have used it previously to probe systems like ionic liquids,[^32^, ^46^] supramolecular gels[^47^, ^48^, ^49^] and also deep eutectic solvents.^[50]^

Herein, we measured the RLS spectra of the DES and of solutions containing the DES and the catalyst conducive to the best yield, as reported in Table 3. For the sake of simplicity, we will discuss the results obtained with the glycol‐ and carboxylic acid‐based DES separately.

**Figure 7:**
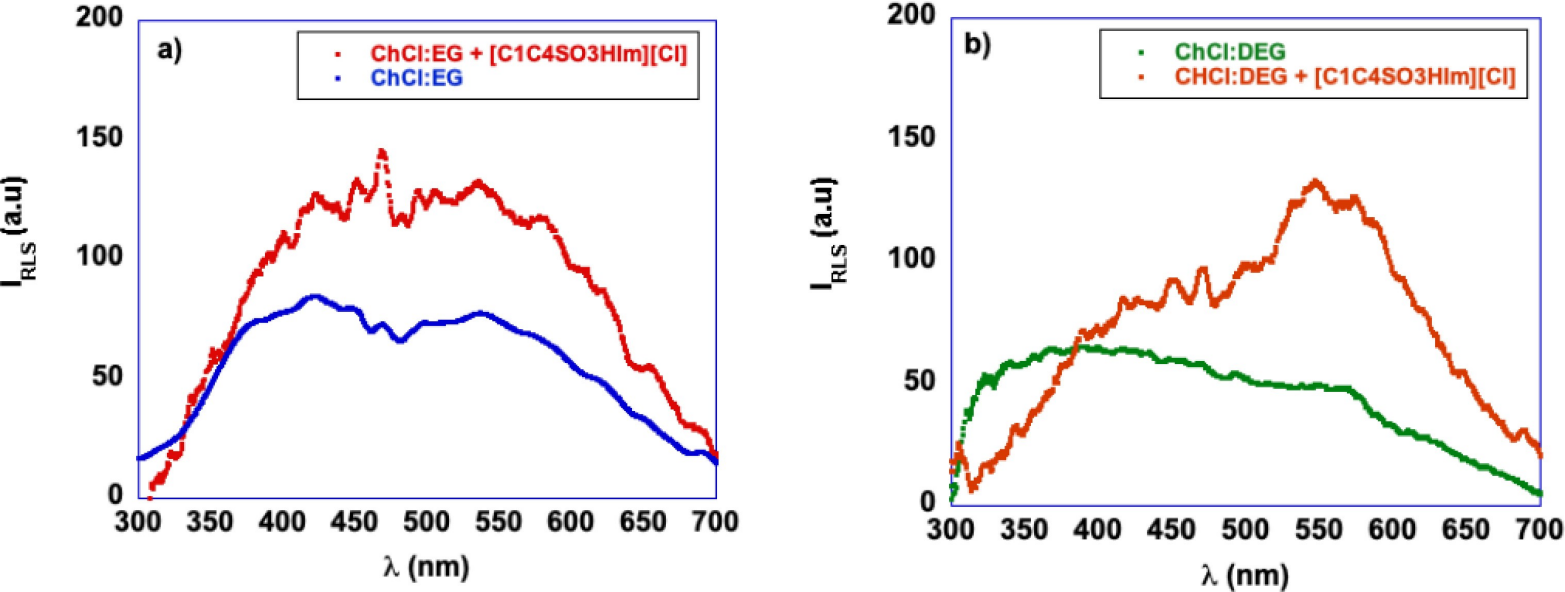
RLS spectra of a) ChCl : EG and ChCl : EG+[C_1_C_4_SO_3_HIm][Cl] and b) ChCl : DEG and ChCl : DEG+[C_1_C_4_SO_3_HIm][Cl].

The RLS spectra obtained for the glycol‐based DES, ChCl : EG and ChCl : DEG, with and without the catalyst, [C_1_C_4_SO_3_HIm][Cl], at the same composition used in the reactions, are reported in Figure 7.

Examining the plots reported in Figure 7a and 7b, an increase in I_RLS_ in the presence of the catalyst can be observed, hinting at a major solvent reorganization, likely due to the occurrence of additional hydrogen bonds involving the HBD and HBA.

**Figure 8:**
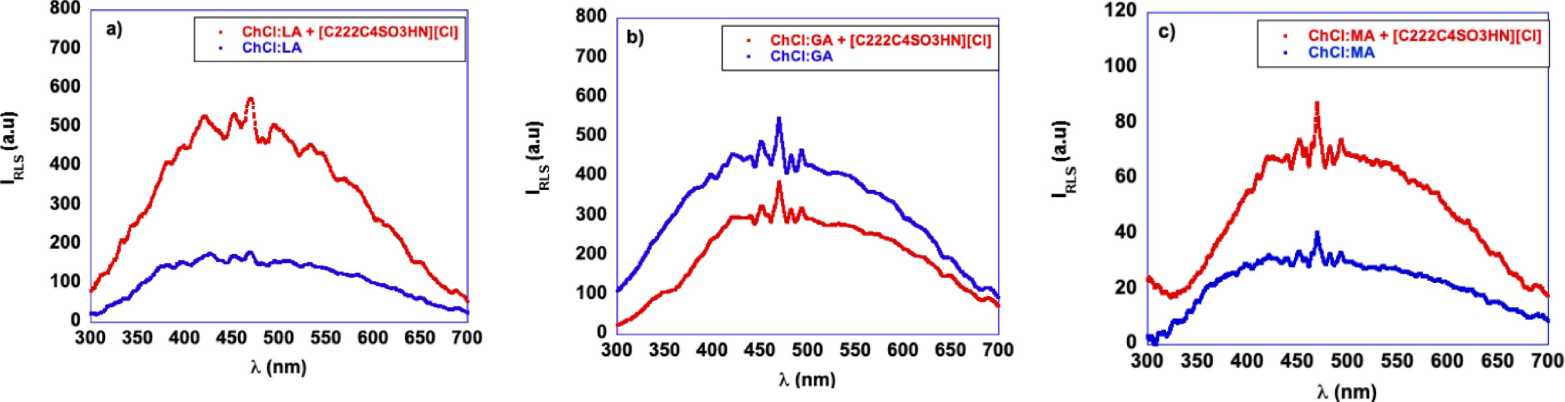
RLS spectra of a) ChCl : LA and ChCl : LA+[C_222_C_4_SO_3_HN][Cl], b) ChCl : GA and ChCl : GA+[C_222_C_4_SO_3_HN][Cl], and c) ChCl : MA and ChCl : MA+[C_222_C_4_SO_3_HN][Cl].

With regard to the same investigation in the carboxylic acid‐based DES, the RLS spectra with and without the catalyst [C_222_C_4_SO_3_HN][Cl] are reported in Figure 8. It is important to consider that in the case of the solutions in ChCl : MA, we could not record the spectra with the same settings used for all the other DES, since the intensity was beyond the instrument limit. Therefore, the spectra reported in Figure 8c were recorded using shorter excitation and emission bandwidths.

Examination of the plots reported in Figure 8, points out a different behavior in these DES. In particular, upon adding the catalyst, we observe a significant rise in I_RLS_ suggesting more extended aggregation compared with the neat DES for ChCl : LA, and ChCl : MA, featuring monocarboxylic acids as HBD. Conversely, in solution of the dicarboxylic acid‐based DES, ChCl : GA, a slight reduction in I_RLS_ occurs. If we compare the trends of I_RLS_ in the presence of the catalyst, we observe that, in general, higher yields were detected in solvent systems in which the catalyst induced a solvent reorganization, giving rise to the presence of more extended aggregates. Interestingly, this phenomenon occurs in DES of lower viscosity, and it is more significant in the presence of aromatic than aliphatic catalysts.

### Optimization of Catalyst Loading, Scale‐Up and Recycling

**Figure 9:**
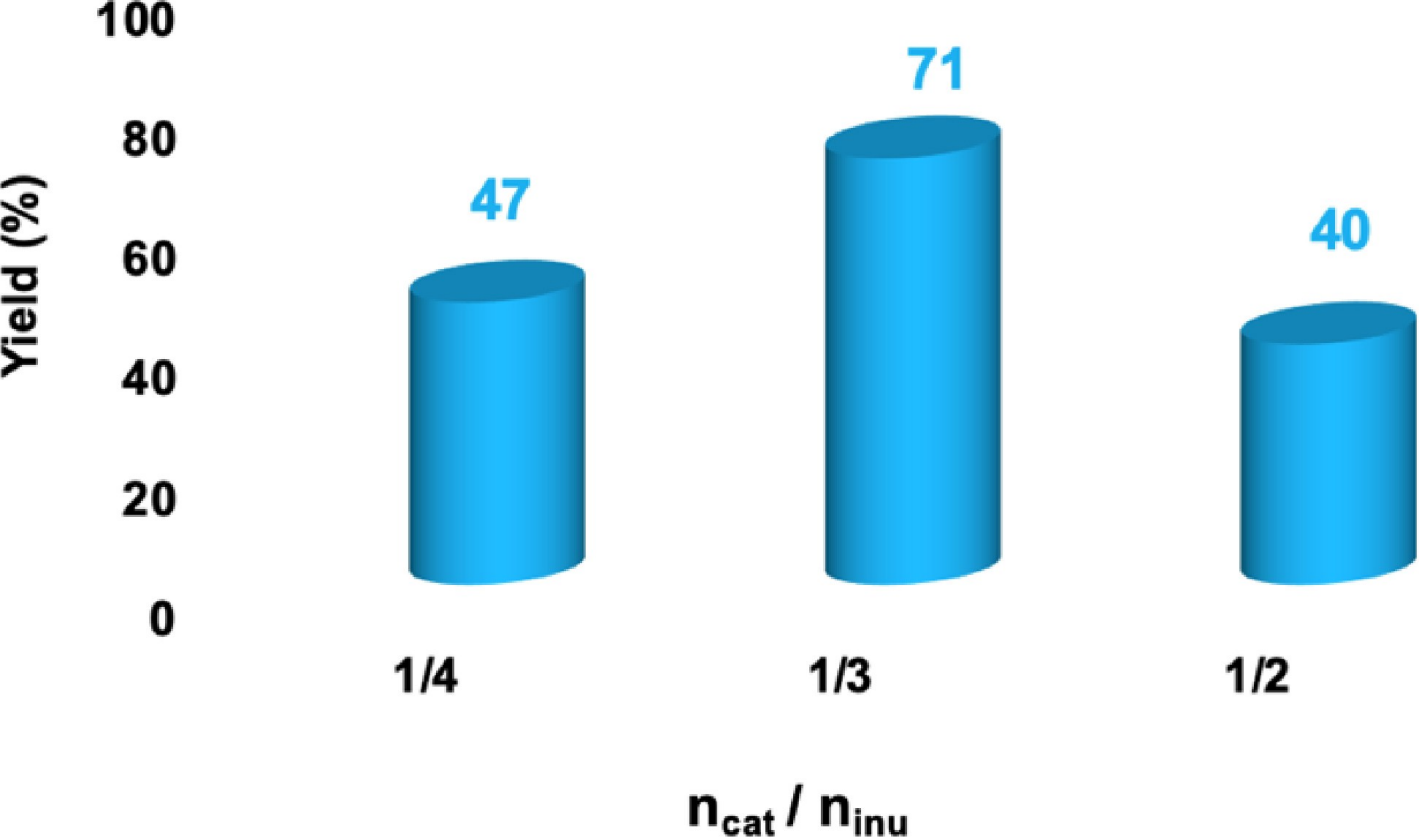
Comparison of the yields in 5‐HMF obtained at 80 °C, 3 h, in the presence of [C_1_C_4_SO_3_HIm][Cl], in ChCl : EG, at varying catalyst/substrate molar ratios.

Given that the best yield was observed when conducting the reaction in the presence of [C_1_C_4_SO_3_HIm][Cl], in solution of ChCl : EG, we used this system to optimize a further experimental parameter, such as the catalyst loading. To this end, we carried out the inulin to 5‐HMF conversion at various molar ratios between catalyst and inulin from 1/4 to 1/2, obtaining the results reported in Figure 9.

Figure 9 clearly shows that an increase in catalyst loading initially favours the reaction up to a ratio of 1/3, after which a further increase in catalyst amount results in a decreased yield in 5‐HMF, due acid‐catalysed degradation processes.

Then, to further prove the suitability of our protocol, we carried out the process, under optimized condition, by increasing ten‐fold the initial amount of inulin. The yield obtained was 49 %, lower than that obtained with 20 mg of inulin (71 %), likely due to more difficult mass transfer.

**Figure 10:**
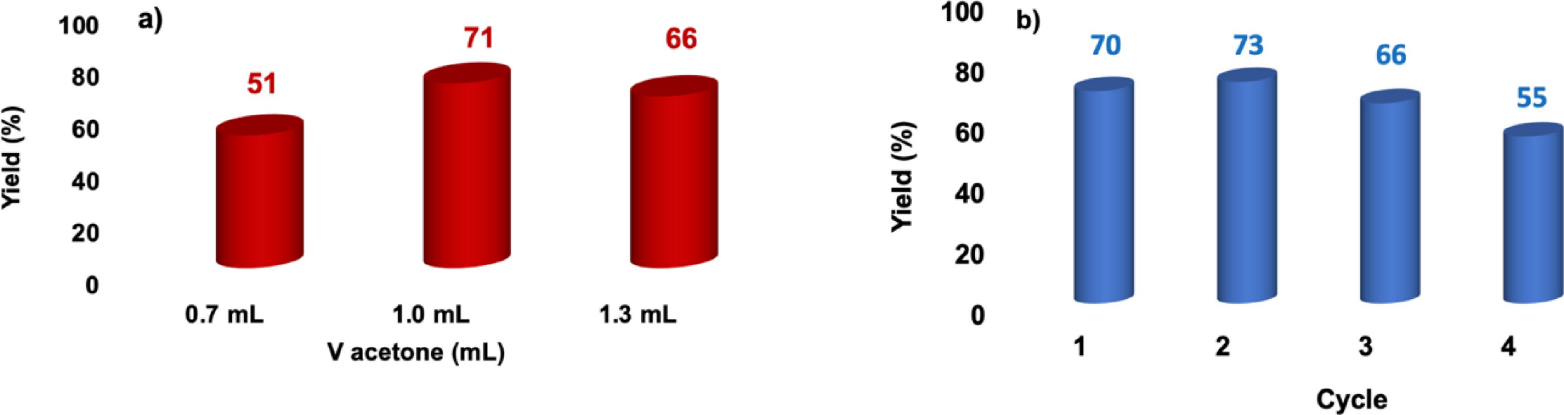
a) Yields in 5‐HMF obtained at 80 °C, 3 h, in the presence of [C_1_C_4_SO_3_HIm][Cl], in ChCl : EG, at varying volumes of acetone as extracting solvent and b) yields obtained in recycling tests.

Another parameter that we wanted to optimize is the amount of acetone as extracting solvent. We therefore conducted the reaction in ChCl : EG, in the presence of [C_1_C_4_SO_3_HIm][Cl], employing different volumes of acetone, ranging from 0.7 to 1.3 mL, for 250 mg of DES, under the same experimental conditions described above. The results obtained, reported in Figure 10a, clearly show that 1 mL is the optimal volume of acetone, as no obvious improvement in yields occurs when increasing the volume of extracting solvent to 1.3 mL.

Finally, to assess the recyclability of the catalyst and the DES, we evaluated the possibility of reusing the best‐performing catalyst, [C_1_C_4_SO_3_HIm][Cl], in ChCl : EG. After the first reaction, the acetone layer was removed, and the DES was charged with a fresh batch and 1 mL of acetone. This procedure was repeated until a loss in yield higher than 10 % was observed. The results obtained are reported in Figure 8b. These results show a good recyclability of the system, which was reused 3 times without significant loss in yields. It is also worth noting that no extraction step is required in between two consecutive runs, and both the catalyst and the solvent are recycled.

To summarize, the results obtained in this work shows that under optimized conditions, our catalytic system, consisting of [C_1_C_4_SO_3_HIm][Cl] in solution of ChCl : EG, in the presence of acetone as extracting solvent, at 80 °C, enables conversion of inulin to 5‐HMF, with a 71 % yield, and can be reused 3 times without significant loss in yield.

To better evaluate the performance of our system, we compare the yield and reaction conditions observed in this work with those reported in literature, dealing with the same transformation. These are reported in Table 4.

**Table 4:** Comparison of our best results with other systems reported in the literature.

Entry	Solvent	Catalyst	T (°C)	T (h)	Yield in 5‐HMF (%)
1	ChCl : EG^a^	[C_1_C_4_SO_3_HIm][Cl]	80	3	71
2	ChCl : EG/H_2_O^[51]^	CO_2_	120	6	38
3	H_2_O/1‐butanol^[52]^	TiO_2_/mordenite	160	1	61
4	[C_1_C_4_SO_3_HIm][HSO_4_]^[53]^	[C_1_C_4_SO_3_HIm][HSO_4_]	100	1	62
5	[C_1_C_2_Im][Cl]^[54]^	Supported MOF	120	6	72
6	[C_1_C_4_Im][Cl]^[55]^	Carbonaceous microspheres	80	1	59
7	DMSO^[56]^	Niobium phosphotungstate	80	3	53
8	DMSO/H_2_O^[57]^	Silica nanoparticles	120	3	87
9	[C_1_C_4_Im][Cl]^[26]^	Lignosulfonic acid	100	0.16	94
10	DMSO^[58]^	Sulfated mesoporous Nb_2_O_5_	110	5	60
11	DMSO^[59]^	SnCl_4_‐TBAB	140	2	62
12	[C_1_C_4_Im][Cl]^[60]^	[C_1_C_4_Im][HSO_4_]	80	1.08	82
13	H_2_O^[61]^	CO_2_ 6 MPa	180	1.5	50
14	[C_1_C_4_Im][HSO_4_]^[62]^	H_3_BO_3_‐SiO_2_	120	5	88

[a] This work.

Examination of the results reported in Table 4, clearly shows that our catalytic system is competitive with most of the ones so far reported in literature for the conversion of inulin into 5‐HMF. In particular, our protocol leads to a higher yield in 5‐HMF, using milder conditions compared with several systems employing CO_2_ in a diluted DES,^[51]^ zeolite‐TiO_2_ composites,^[52]^ and also the acidic IL [C_1_C_4_SO_3_HIm][HSO_4_], as both solvent and catalyst,^[53]^ (entries 1–4). In addition, we achieved the same yield obtained with a supported MOF,^[54]^ but with the advantage of a shorter reaction time and lower temperature (3 h vs 6 h and 80 °C vs 120 °C, respectively, entries 1,5). Likewise, we also obtained higher yields in 5‐HMF, compared with systems working at the same temperature, and employing carbonaceous microspheres,^[55]^ and niobium phosphotungstate^[56]^ (yields=71 %, 59 %, 53 %, respectively, entries 1,6,7). Our systems is conversely outperformed by silica nanoparticles in DMSO/H_2_O,^[57]^ [C_1_C_4_Im][HSO_4_] in [C_1_C_4_Im][Cl],^[60]^ silica supported boric acid^[62]^ and lignosulfonic acid^[26]^ (entries 1,8,9, 12, 14). In this latter case, in particular, a higher yield is achieved in a much shorter time. Finally, the catalytic system described herein achieves a higher yield in 5‐HMF compared with Nb (V) oxides,^[58]^ a mixture of the Lewis acidic SnCl_4_ and ammonium salts,^[59]^ or CO_2_ under high pressure,^[61]^ with the added benefit of using a lower temperature (yield=71 %, 60 %, 62 % and 50 %, respectively, entries 1, 10–12).

## Conclusions

In this work, we studied the dehydration of inulin into 5‐hydroxymethylfurfural in deep eutectic solvents, employing acidic task‐specific ionic liquids as catalyst, in a biphasic system featuring acetone as extracting solvent, which allowed the continuous extraction of the reaction product. We optimized the experimental conditions, achieving a 71 % of yield, in ChCl : EG, in the presence of [C_1_C_4_SO_3_HIm][Cl] as catalyst, at 80 °C, in 3 hours.

We also found that the use of acetone as an extracting phase is necessary to obtain good yields in 5‐HMF, since in its absence 5‐HMF undergoes extensive degradation. In our view, these results are significant because our protocol achieves higher yields in milder conditions with respect to most systems reported in literature for the same reaction.

Notably, we also elucidated some relations between properties of the solvent medium and yields obtained. Higher solvent viscosities are found to be detrimental to the 5‐HMF yield.

Analysis of structural features of the solvents highlights that the presence of the catalysts induces appreciable perturbation in the DES structural organization. This proves to be more relevant in the presence of less viscous solvents and gives rise to the formation of more extended aggregates. Interestingly, the above systems are also the ones in which higher yields can be detected. Finally, regarding the influence exerted by the structure of the catalyst in the reaction outcome, no obvious effect was detected, upon changing the nature of the cationic head. The most significant influence derives from the length of the alkyl spacer. In general, with the cation being the same, the catalyst acidity increases going from a propyl to a butyl spacer and, in the ChCl : EG/[C_1_C_4_SO_3_HIm][Cl] system, this gives rise to the highest yield in 5‐HMF.

## Author Contributions

**Salvatore Marullo**: writing, original draft, visualization; **Giovanna Raia**: investigation; **Josh J. Bailey**: review and editing; **H**. **Q. Nimal Gunaratne**: supervision, review and editing; **Francesca D′Anna**: Supervision, funding acquisition, conceptualization, writing, original draft, review and editing;

## Conflict of Interests

Authors declare no competing financial interest.

1

## Supporting information

As a service to our authors and readers, this journal provides supporting information supplied by the authors. Such materials are peer reviewed and may be re‐organized for online delivery, but are not copy‐edited or typeset. Technical support issues arising from supporting information (other than missing files) should be addressed to the authors.

Supporting Information

## Data Availability

The data that support the findings of this study are available in the supplementary material of this article.
