# Supplementary material for: Inulin Dehydration to 5‐HMF in Deep Eutectic Solvents Catalyzed by Acidic Ionic Liquids Under Mild Conditions
Source: ChemSusChem. 2025 Jan 24;18(10):e202402522. doi: 10.1002/cssc.202402522 (PMC12094151; doi:10.1002/cssc.202402522)
Supplement: Supplementary file 1 — Supporting Information [file CSSC-18-e202402522-s001.pdf]

ChemSusChem

Supporting Information

Inulin Dehydration to 5-HMF in Deep Eutectic Solvents Catalyzed by Acidic Ionic Liquids Under Mild Conditions

Salvatore Marullo, Giovanna Raia, Josh J. Bailey, H. Q. Nimal Gunaratne,* and Francesca D′Anna*

Supporting information for

Inulin Dehydration to 5-HMF in Deep Eutectic Solvents catalyzed by acidic Ionic Liquids Under Mild Conditions

Table of contents

**Figure S1.** TGA traces for the catalysts empolyed.	S1
**Figure S2.** Plots of yield in 5-HMF from inulin at 80 °C, in different DES and in the presence of different catalysts.	S4
**Figure S3.** Plots temperature-dependent viscosity measurements for pure DES and solutions of the catalyst in solution of the DES considered.	S5
**Figure S4.** Plots of yields in 5-HMF, in the presence of TSILs, differing for the spacer length.	S7
**Figure S5.** 1H NMR spectra in D6-DMSOof the reaction mixtures after 2 min and 3 h in ChCl:EG	S8
**Figure S6.** ^{1}H NMR spectrum of 5-HMF isolated.	S12
**Table S1** Yields in 5-HMF for the dehydration of inulin at 80 °C, in ChCl:EG	S13
**Table S2** Yields in 5-HMF for the dehydration of inulin at 80 °C, in ChCl:DEG	S14
**Table S3** Yields in 5-HMF for the dehydration of inulin at 80 °C, in ChCl:LA	S15
**Table S4** Yields in 5-HMF for the dehydration of inulin at 80 °C, in ChCl:GA	S16
**Table S5** Yields in 5-HMF for the dehydration of inulin at 80 °C, in ChCl:MA	S17

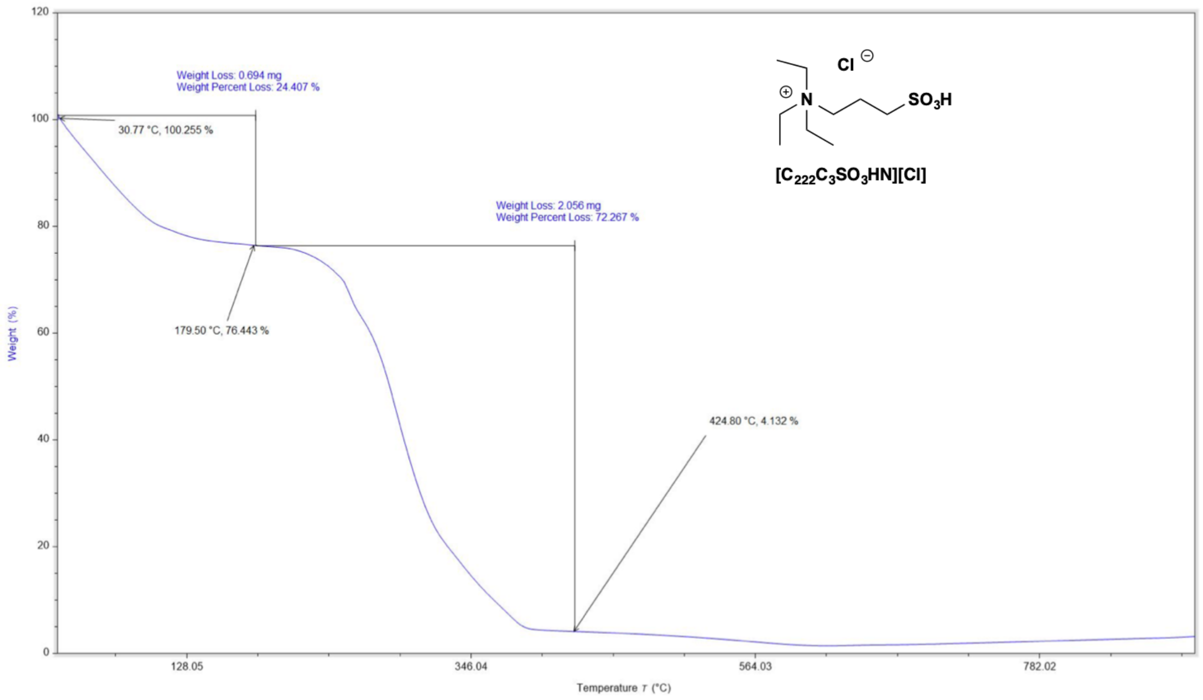
Weight Loss: 0.694 mg
Weight Percent Loss: 24.407 %
30.77 °C, 100.255 %
Weight Loss: 2.056 mg
Weight Percent Loss: 72.267 %
179.50 °C, 76.443 %
424.80 °C, 4.132 %
Weight (%)
Temperature T (°C)
Cl
N
SO3H
[C222C3SO3HN][Cl]

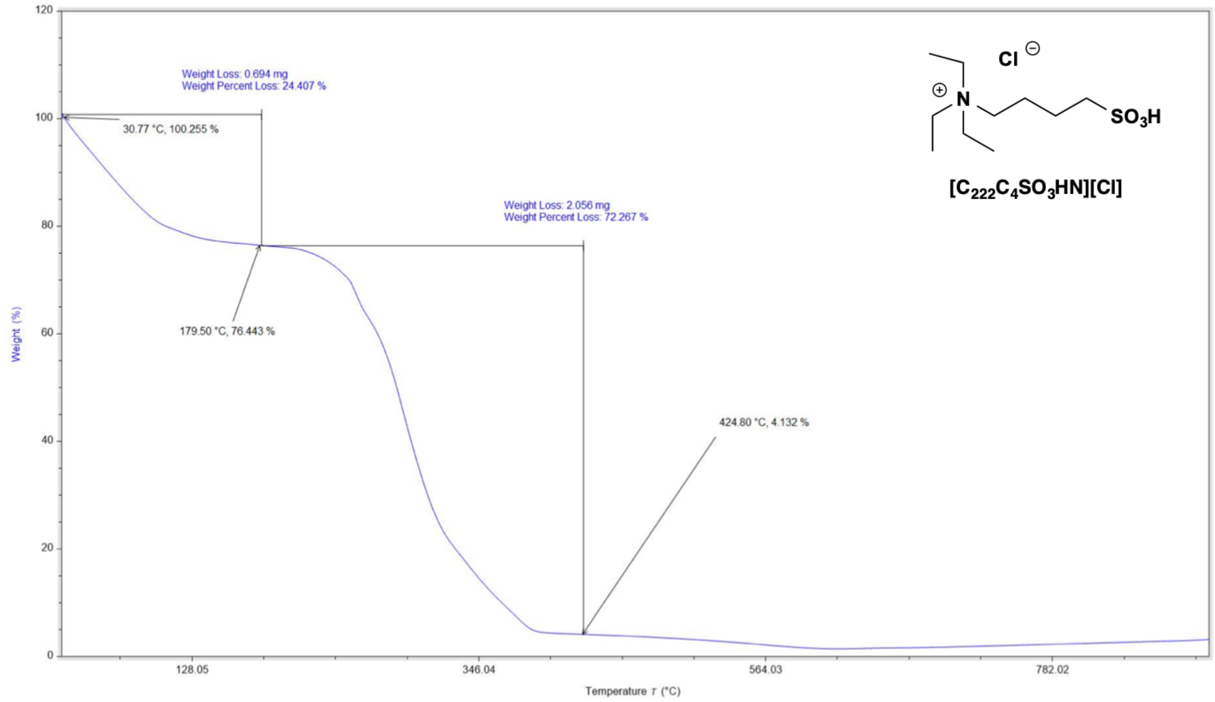
Weight Loss: 0.694 mg
Weight Percent Loss: 24.407 %
30.77 °C, 100.255 %
Weight Loss: 2.056 mg
Weight Percent Loss: 72.267 %
179.50 °C, 76.443 %
424.80 °C, 4.132 %
Weight (%)
Temperature T (°C)
Cl
N
SO3H
[C222C4SO3HN][Cl]

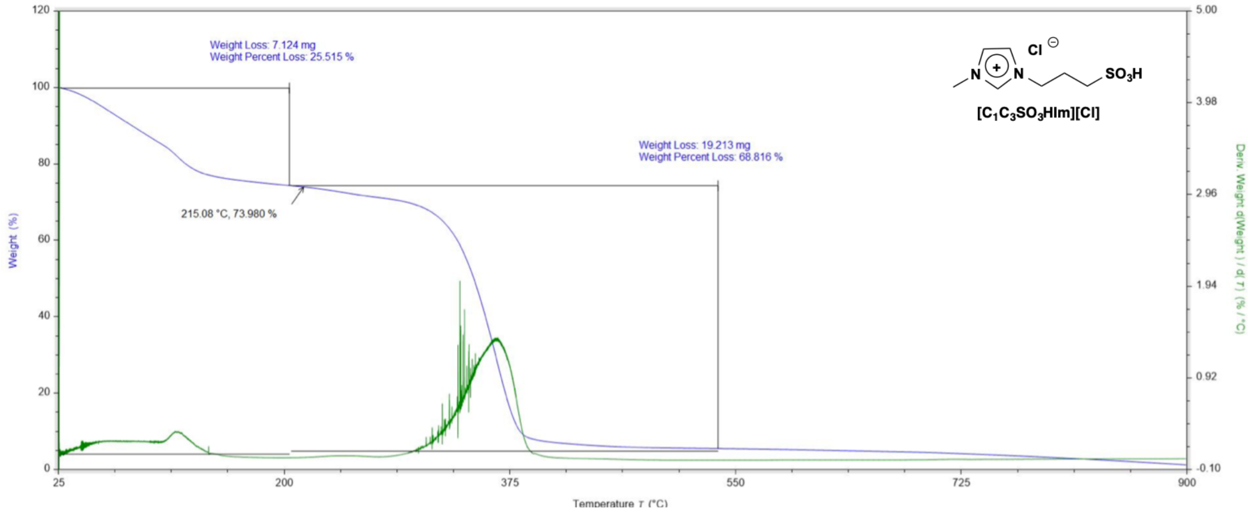

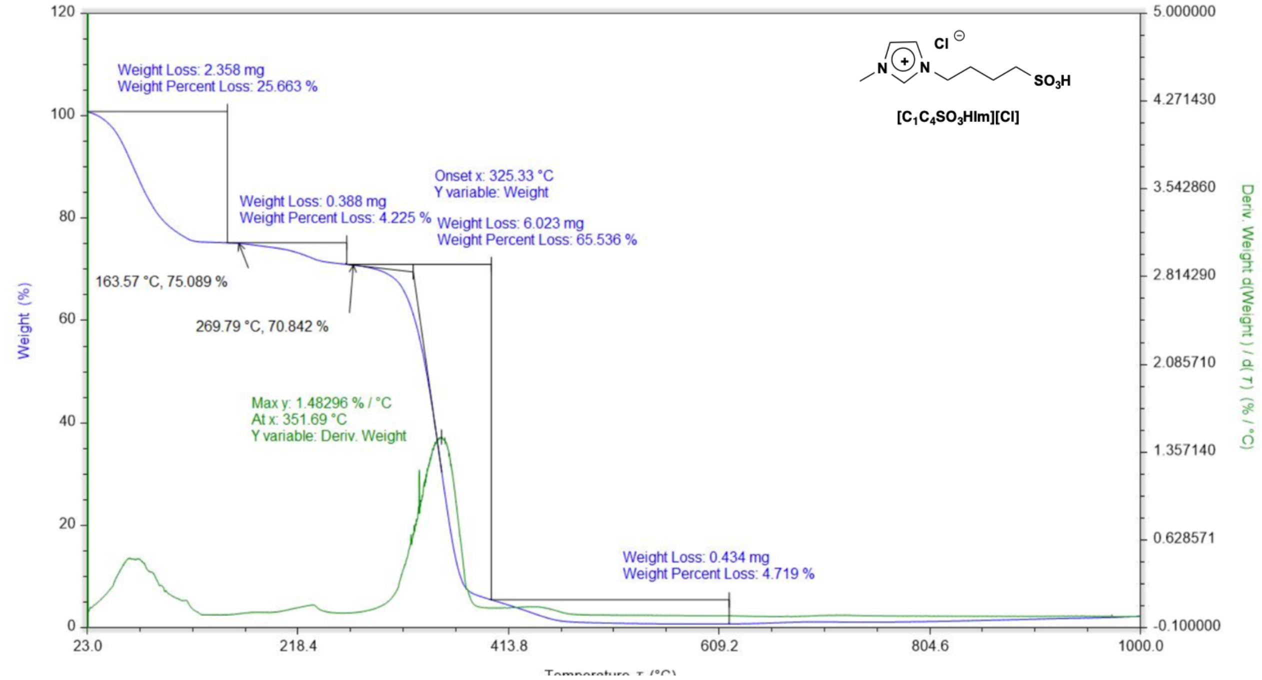

Figure S1. TGA traces for the catalysts empolyed.

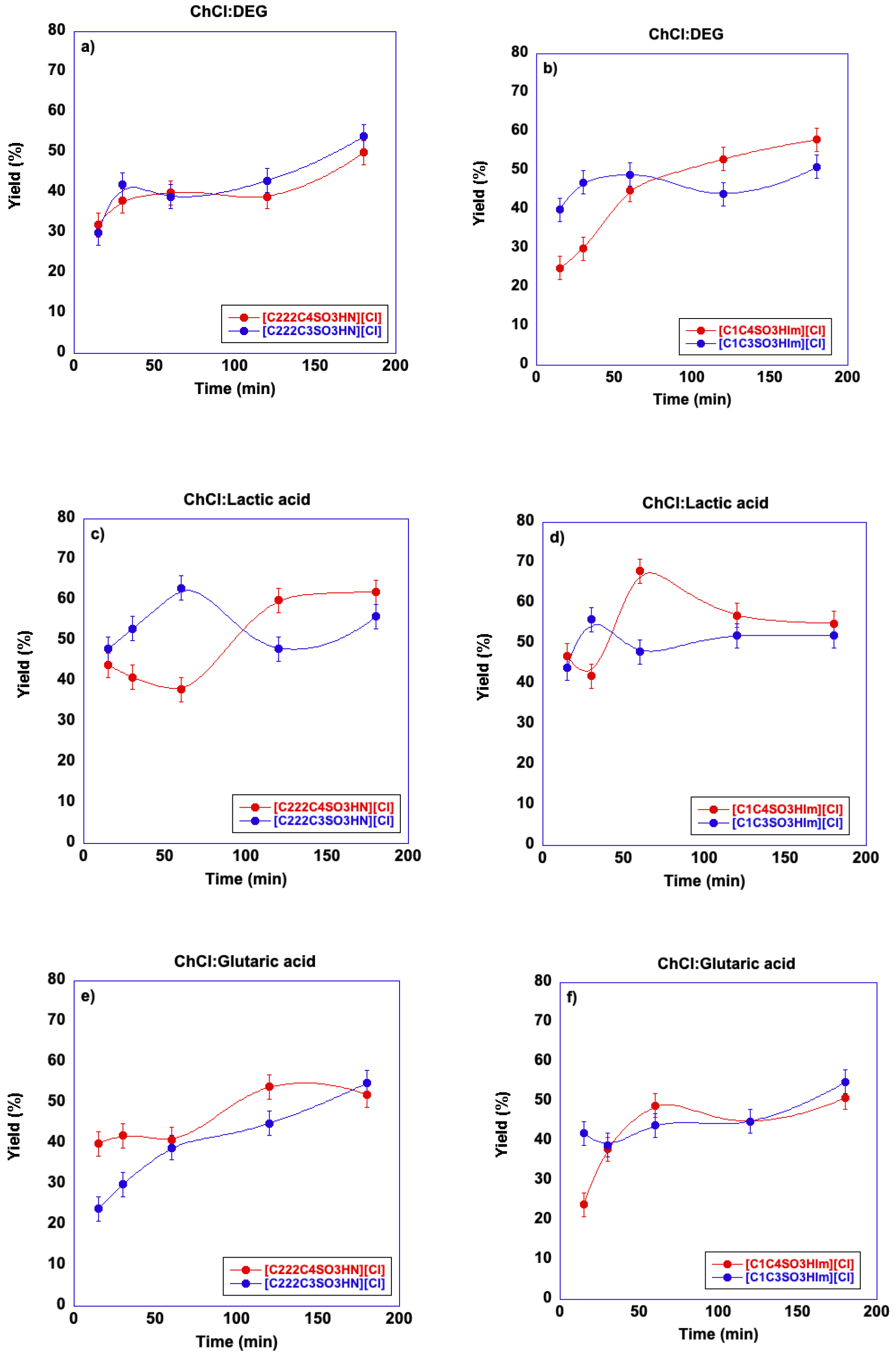
ChCl:DEG
a)
Yield (%)
Time (min)
[C222C4SO3HN][Cl]
[C222C3SO3HN][Cl]
ChCl:DEG
b)
Yield (%)
Time (min)
[C1C4SO3HIm][Cl]
[C1C3SO3HIm][Cl]
ChCl:Lactic acid
c)
Yield (%)
Time (min)
[C222C4SO3HN][Cl]
[C222C3SO3HN][Cl]
ChCl:Lactic acid
d)
Yield (%)
Time (min)
[C1C4SO3HIm][Cl]
[C1C3SO3HIm][Cl]
ChCl:Glutaric acid
e)
Yield (%)
Time (min)
[C222C4SO3HN][Cl]
[C222C3SO3HN][Cl]
ChCl:Glutaric acid
f)
Yield (%)
Time (min)
[C1C4SO3HIm][Cl]
[C1C3SO3HIm][Cl]

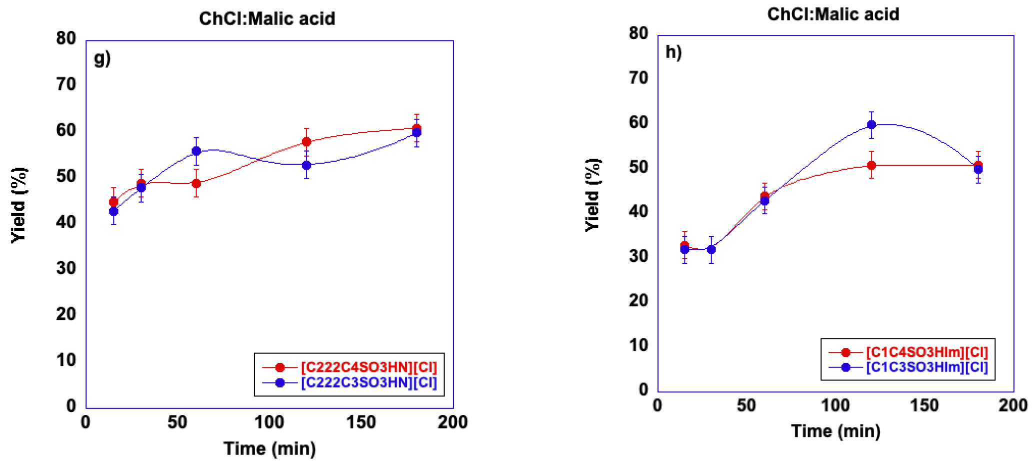

Figure S2. Plots of yield in 5-HMF from inulin at 80 °C, in different DES and in the presence of different acidic TSILs as catalysts.

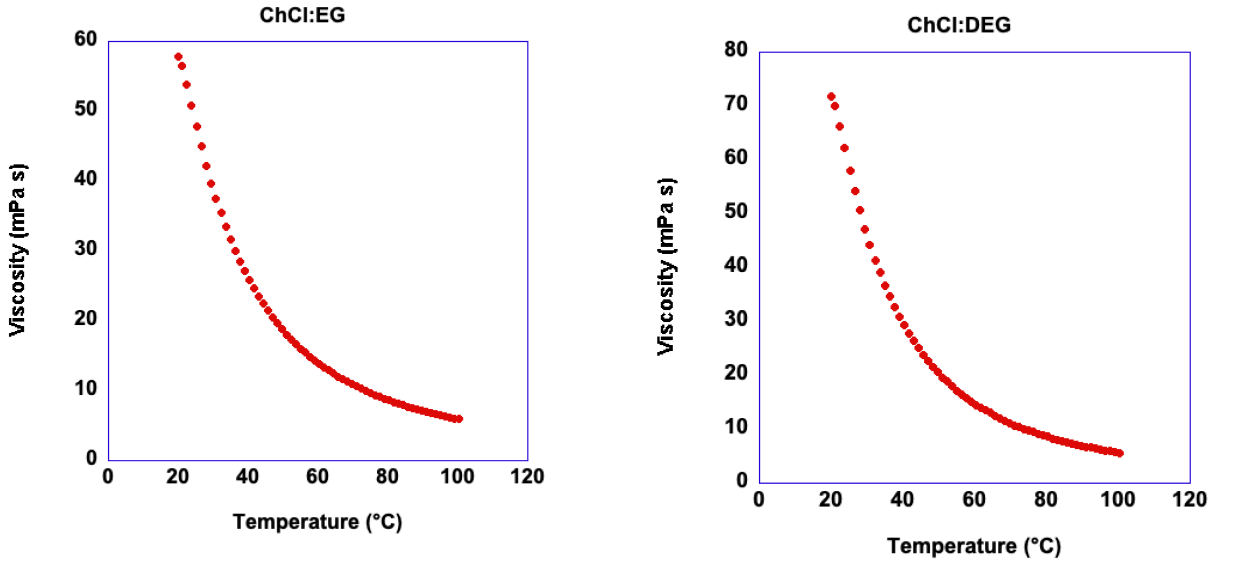

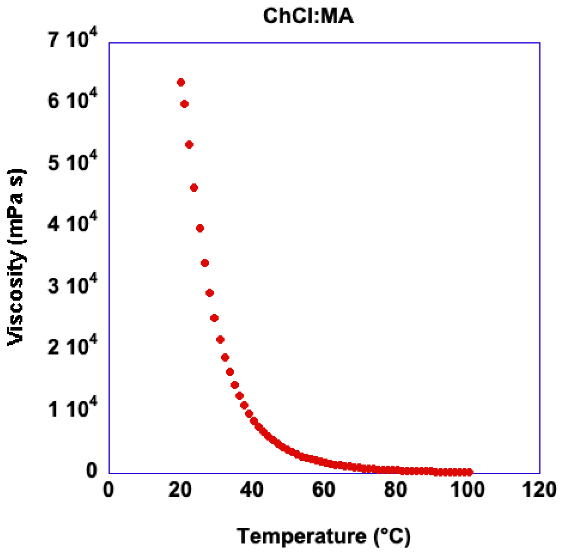
ChCl:MA
Viscosity (mPa s)
Temperature (°C)

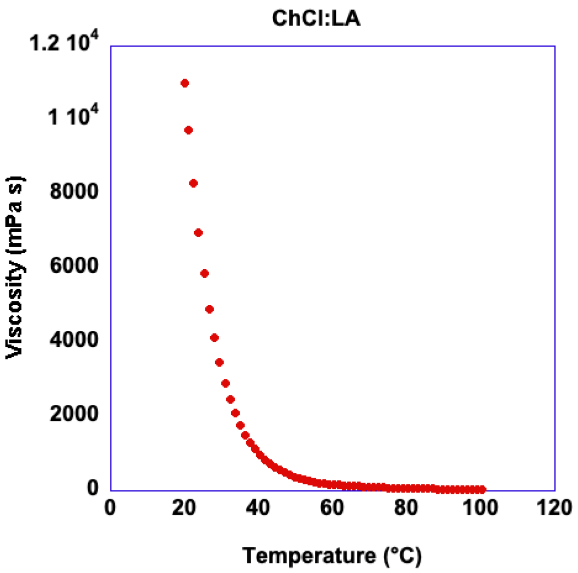
ChCl:LA
Viscosity (mPa s)
Temperature (°C)

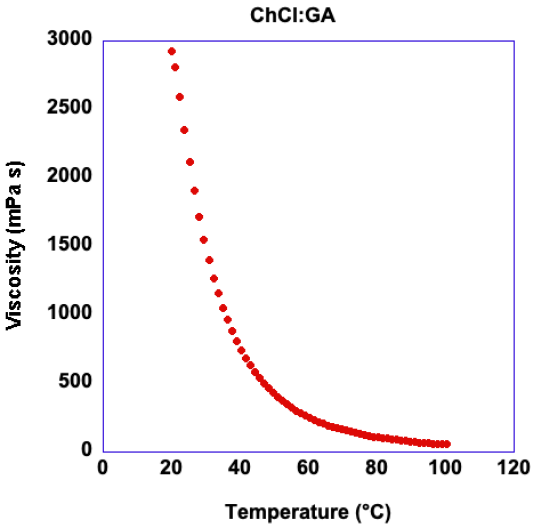
ChCl:GA
Viscosity (mPa s)
Temperature (°C)

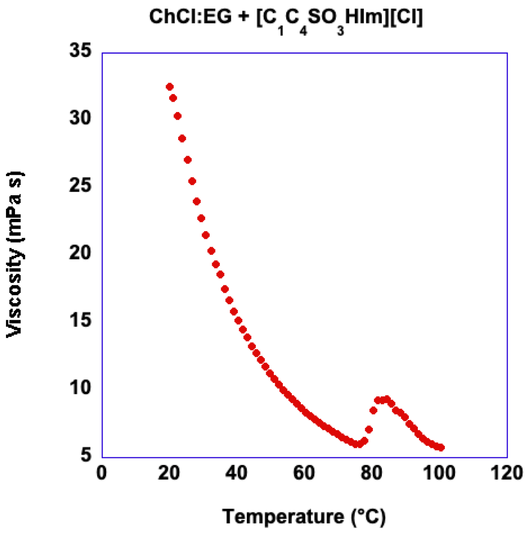
ChCl:EG + [C1C4SO3HIm][Cl]
Viscosity (mPa s)
Temperature (°C)

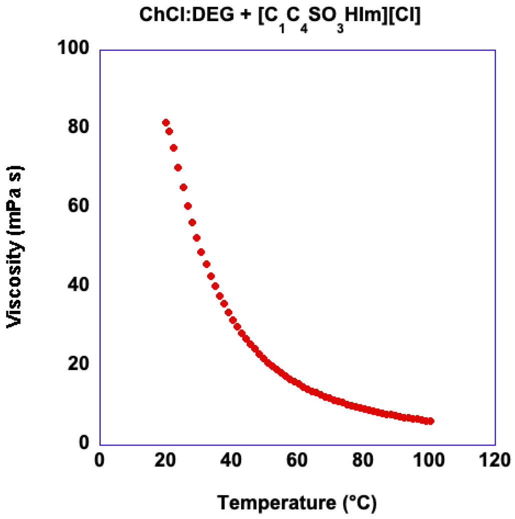
ChCl:DEG + [C1C4SO3HIm][Cl]
Viscosity (mPa s)
Temperature (°C)

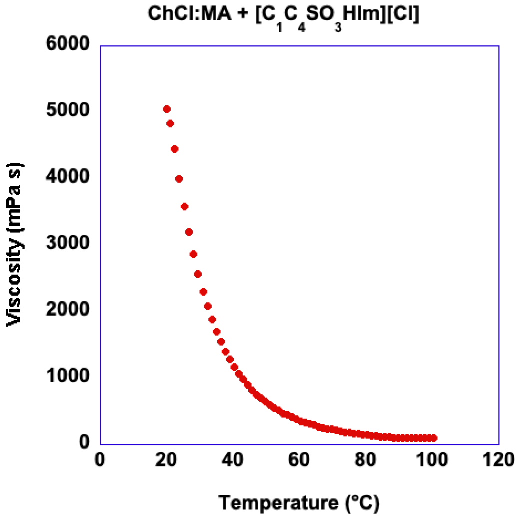
ChCl:MA + [C1C4SO3HIm][Cl]
Viscosity (mPa s)
Temperature (°C)

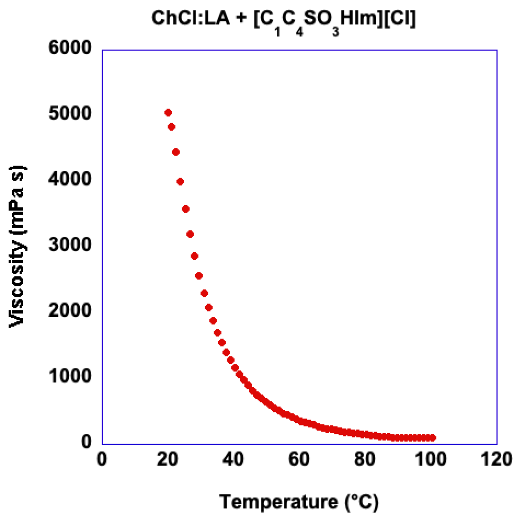

Figure S3. Plots temperature-dependent viscosity measurements for pure DES and solutions of the catalyst in solution of the DES considered.

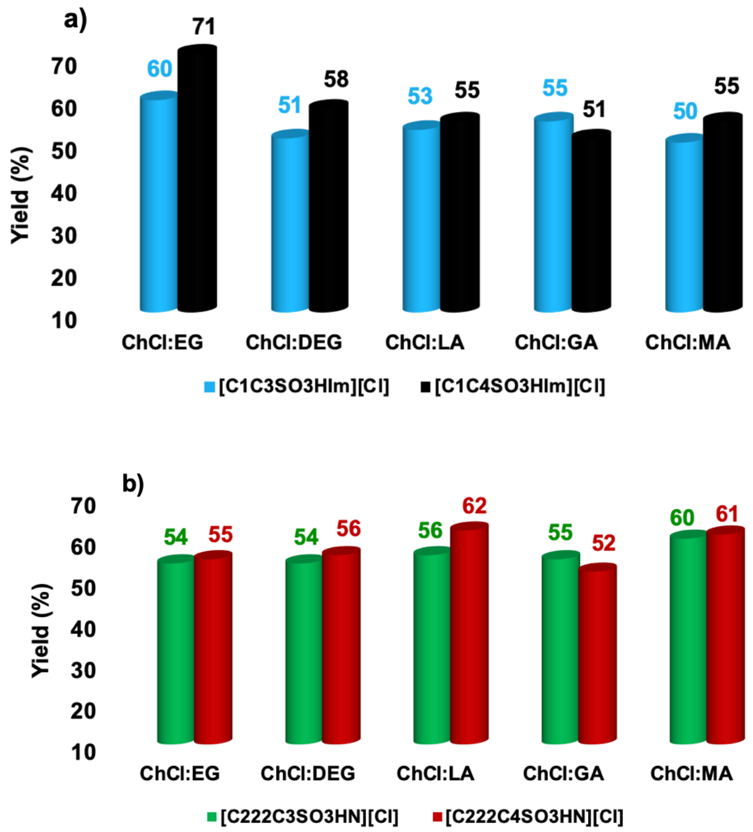

Figure S4. Plots of yields in 5-HMF obtained at 80 °C, 3h, in the presence of a) imidazolium- and b) ammonium-based TSILs, differing for the spacer length.

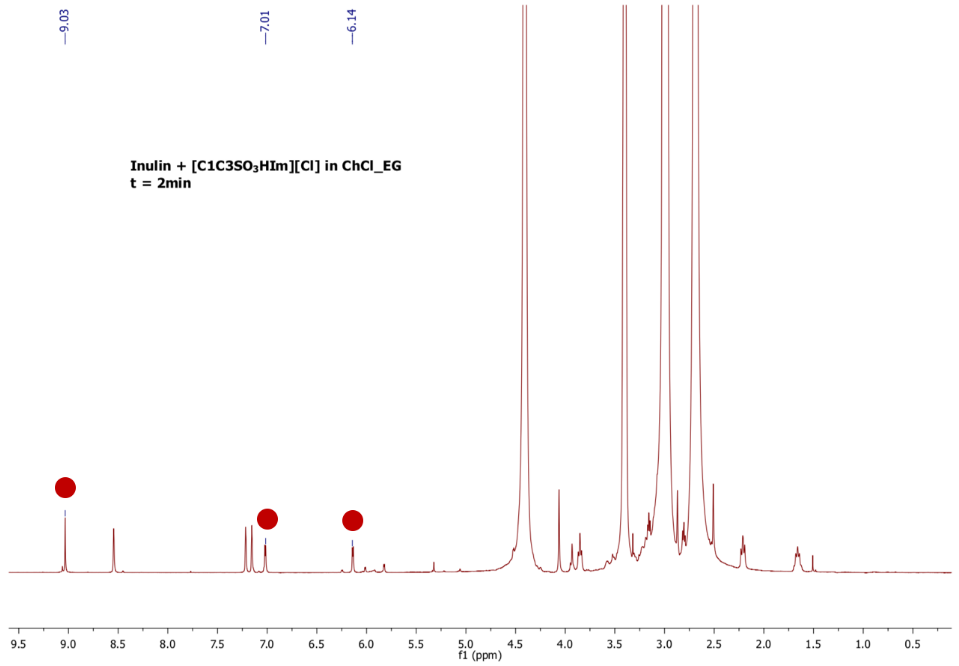

9.03
7.01
6.14
Inulin + [C1C3SO$_3$HIm][Cl] in ChCl_EG
t = 2min
f1 (ppm)

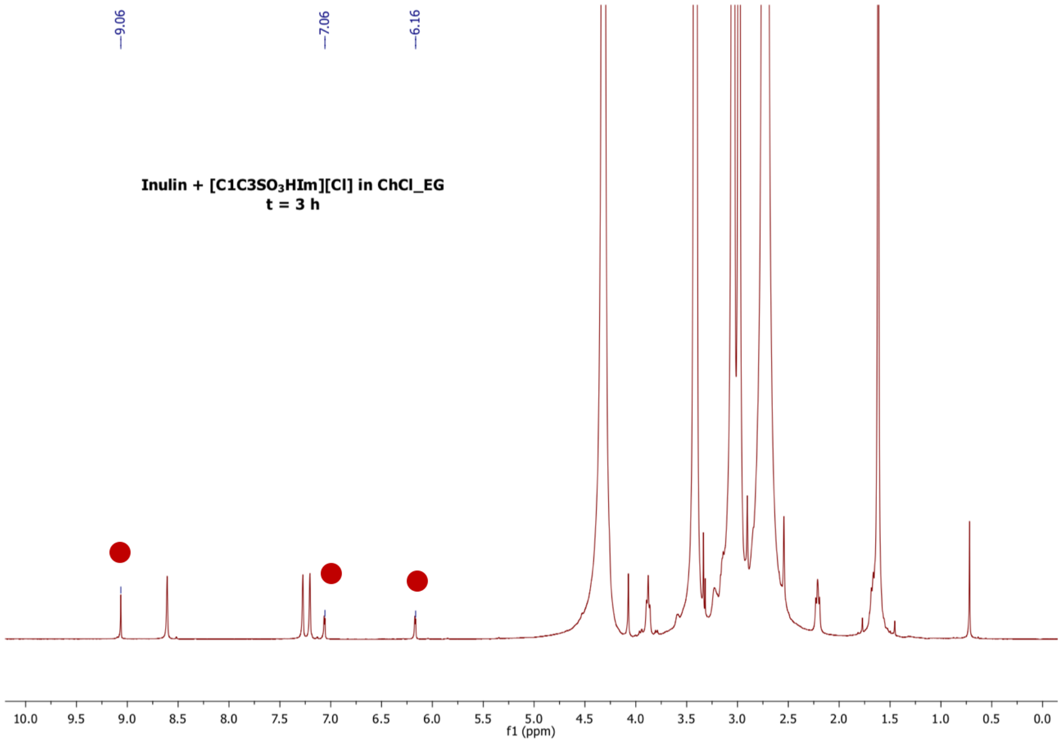

9.06
7.06
6.16
Inulin + [C1C3SO$_3$HIm][Cl] in ChCl_EG
t = 3 h
f1 (ppm)

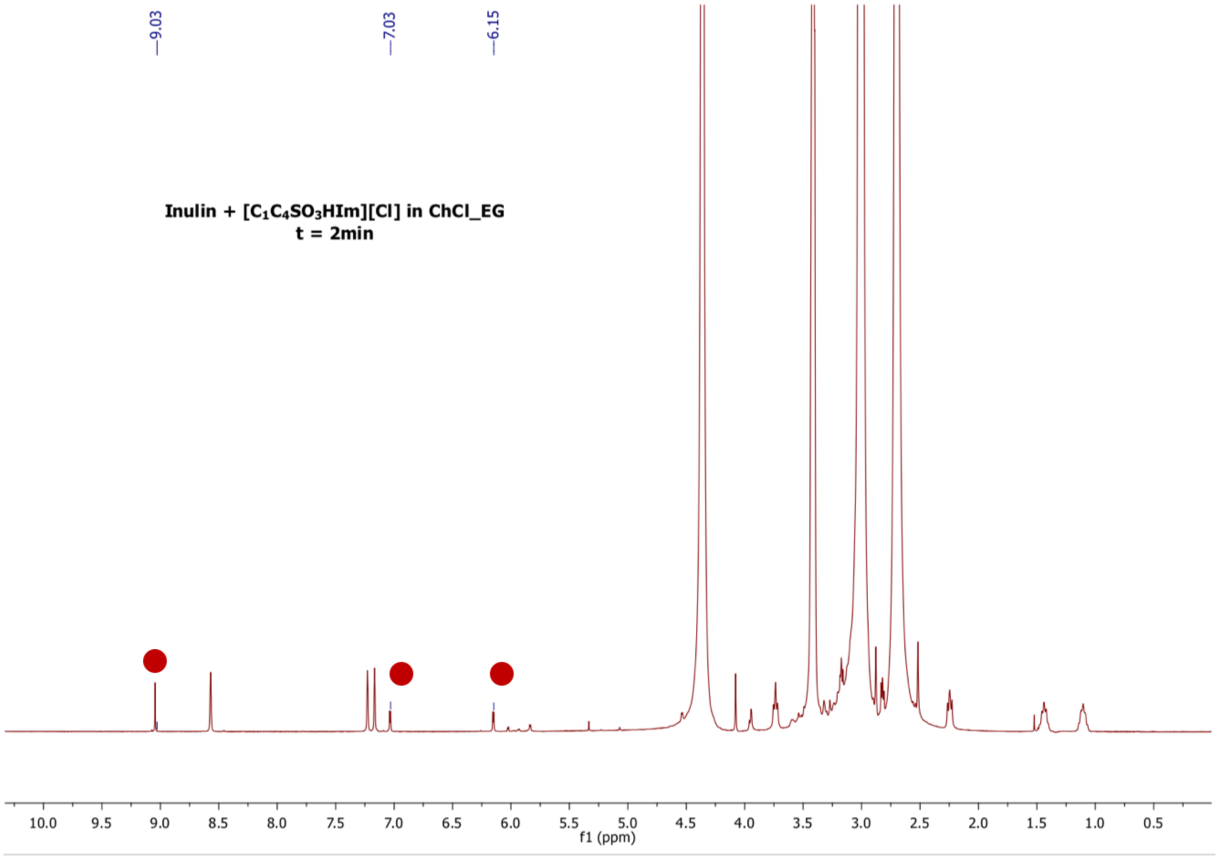

9.03
7.03
6.15
Inulin + [C1C4SO3HIm][Cl] in ChCl_EG
t = 2min
10.0 9.5 9.0 8.5 8.0 7.5 7.0 6.5 6.0 5.5 5.0 4.5 4.0 3.5 3.0 2.5 2.0 1.5 1.0 0.5
f1 (ppm)

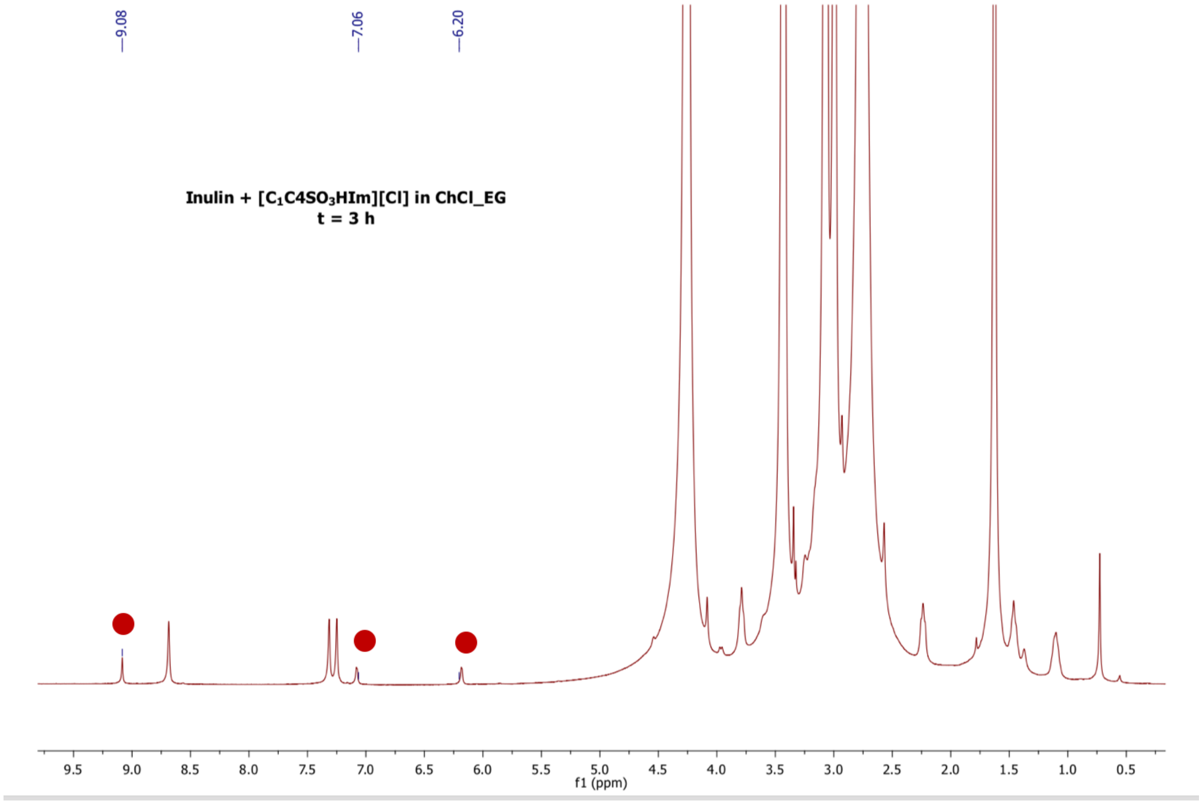

9.08
7.06
6.20
Inulin + [C1C4SO3HIm][Cl] in ChCl_EG
t = 3 h
9.5 9.0 8.5 8.0 7.5 7.0 6.5 6.0 5.5 5.0 4.5 4.0 3.5 3.0 2.5 2.0 1.5 1.0 0.5
f1 (ppm)

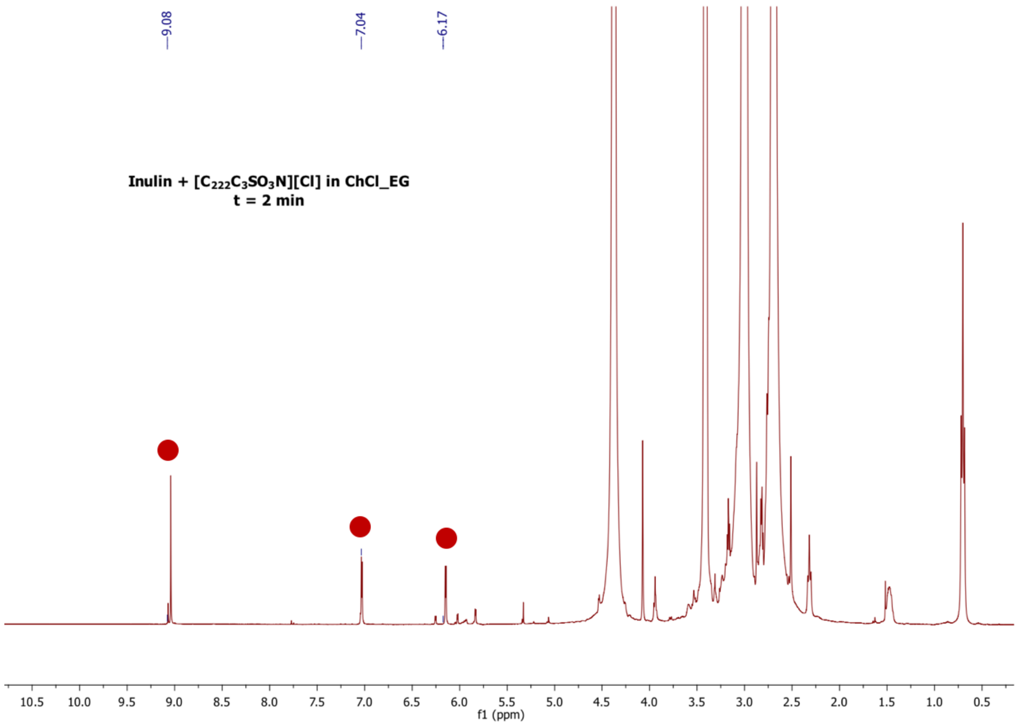

9.08
7.04
6.17
Inulin + [C222C3SO3N][Cl] in ChCl_EG
t = 2 min
10.5 10.0 9.5 9.0 8.5 8.0 7.5 7.0 6.5 6.0 5.5 5.0 4.5 4.0 3.5 3.0 2.5 2.0 1.5 1.0 0.5
f1 (ppm)

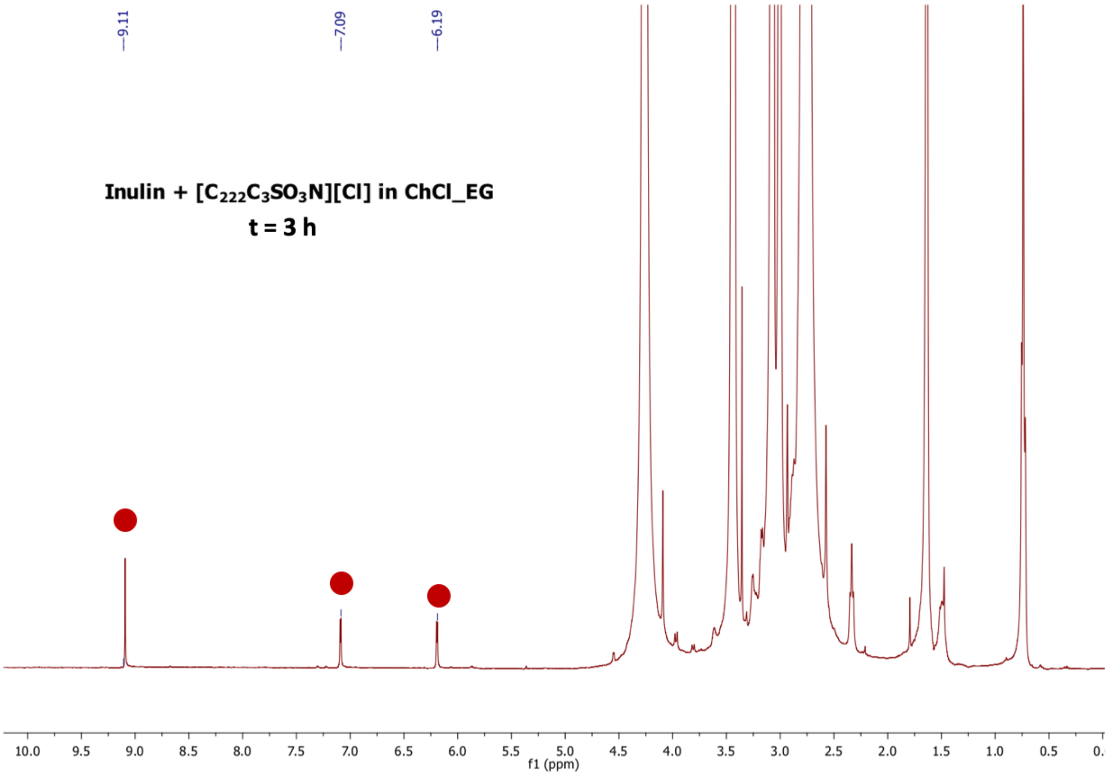

9.11
7.09
6.19
Inulin + [C222C3SO3N][Cl] in ChCl_EG
t = 3 h
10.0 9.5 9.0 8.5 8.0 7.5 7.0 6.5 6.0 5.5 5.0 4.5 4.0 3.5 3.0 2.5 2.0 1.5 1.0 0.5
f1 (ppm)

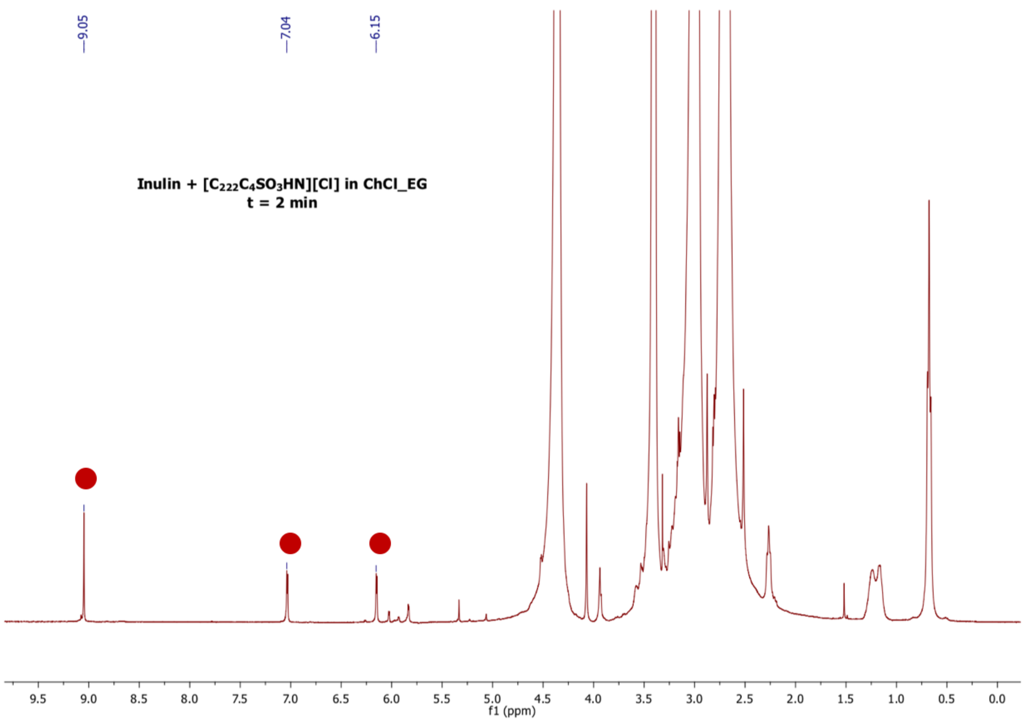

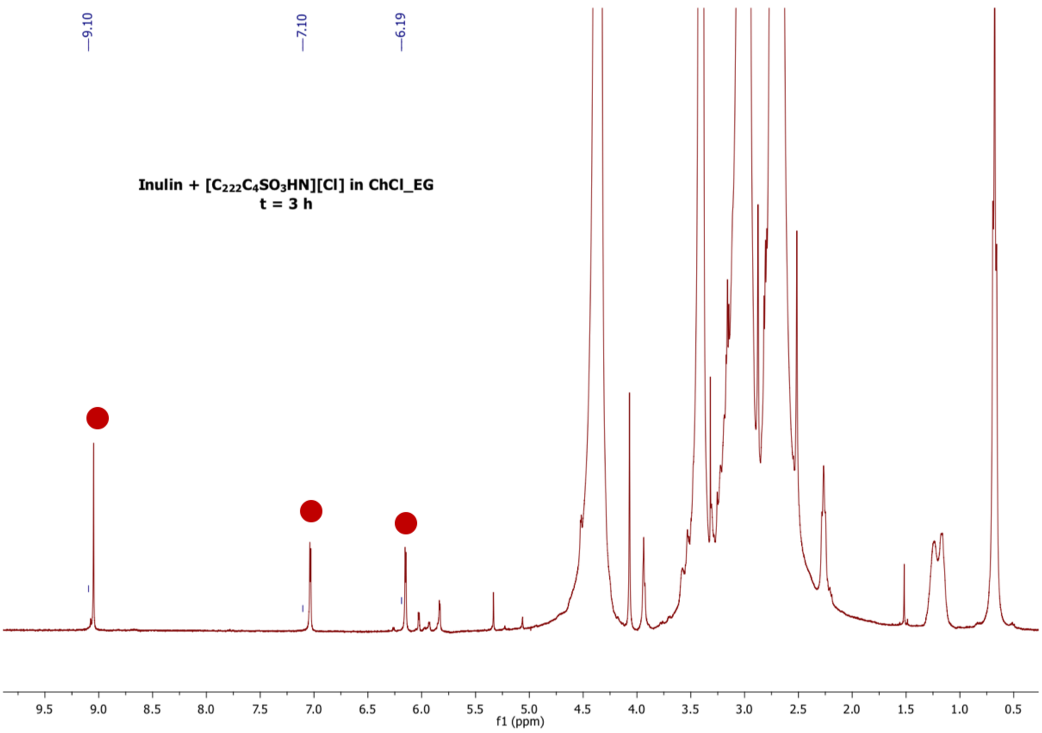

Figure S5. ^{1}H NMR spectra in D6-DMSO of the reaction mixtures after 2 min and 3 h in ChCl:EG. Marked signals refer to protons of 5-HMF

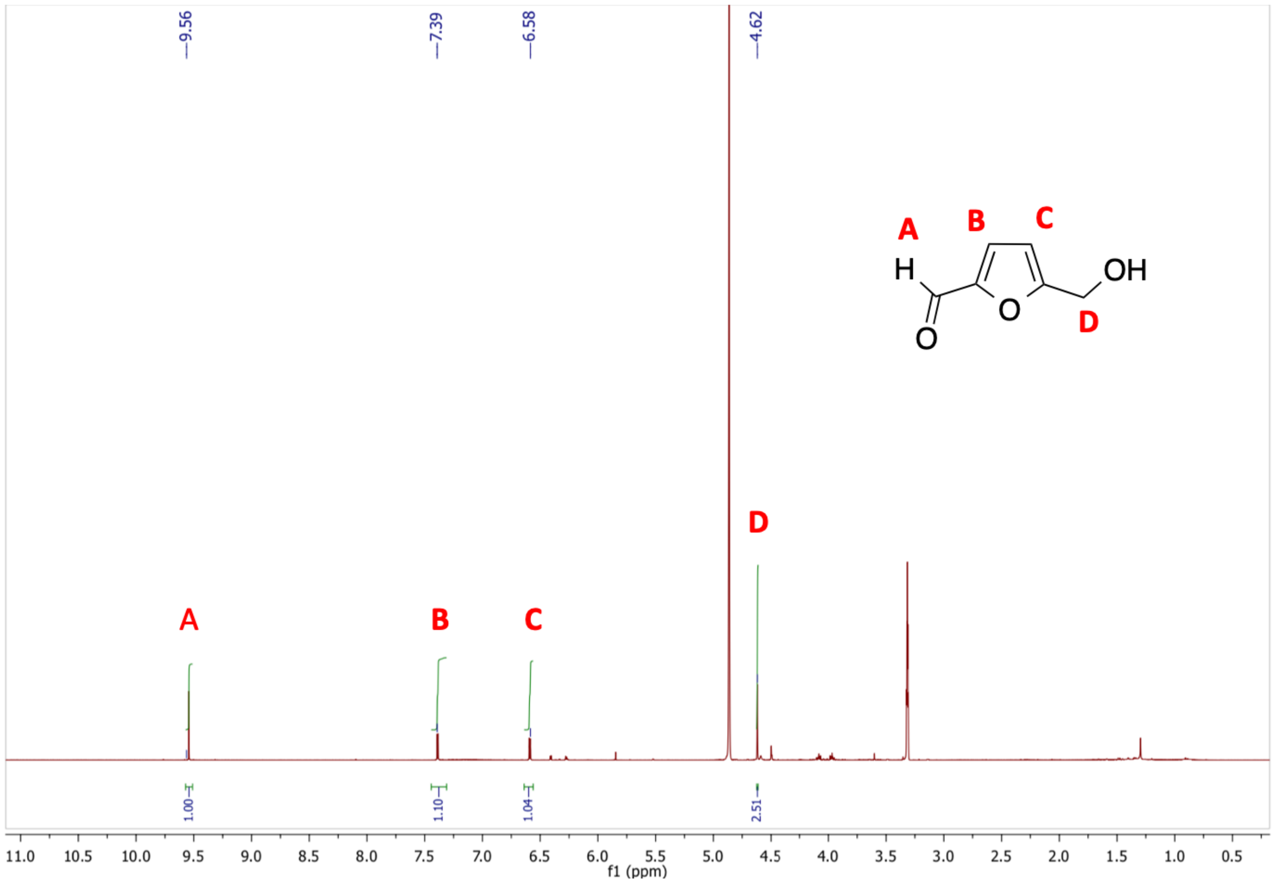

Figure S6. ^{1}H NMR spectrum in CD_3OD of 5-HMF isolated.

Table S1. Yields in 5-HMF for the dehydration of inulin at 80 °C, in ChCl:EG in the presence of acidic TSILs.[a]

Time (min)	Yield in 5-HMF (%)
**$[C_{222}C_3SO_3HN][Cl]$**	
15	37
30	43
60	47
120	44
180	54
**$[C_{222}C_4SO_3HN][Cl]$**	
15	41
30	39
60	50
120	54
180	55
**$[C_1C_3SO_3HIm][Cl]$**	
15	49
30	44
60	57
120	60
180	60
**$[C_1C_4SO_3HIm][Cl]$**	
15	37
30	43
60	47
120	50
180	71

[a] Yields are reproducible within ± 4%.

Table S2. Yields in 5-HMF for the dehydration of inulin at 80 °C, in ChCl:DEG in the presence of acidic TSILs.[a]

Time (min)	Yield in 5-HMF (%)
**$[C_{222}C_3SO_3HN][Cl]$**	
15	30
30	42
60	30
120	43
180	54
**$[C_{222}C_4SO_3HN][Cl]$**	
15	32
30	38
60	40
120	39
180	50
**$[C_1C_3SO_3HIm][Cl]$**	
15	40
30	47
60	49
120	44
180	51
**$[C_1C_4SO_3HIm][Cl]$**	
15	25
30	30
60	45
120	53
180	58

[a] Yields are reproducible within ± 4%.

Table S3. Yields in 5-HMF for the dehydration of inulin at 80 °C, in ChCl:LA in the presence of acidic TSILs.[a]

Time (min)	Yield in 5-HMF (%)
**$[C_{222}C_3SO_3HN][Cl]$**	
15	48
30	53
60	63
120	48
180	56
**$[C_{222}C_4SO_3HN][Cl]$**	
15	44
30	41
60	38
120	60
180	62
**$[C_1C_3SO_3HIm][Cl]$**	
15	44
30	56
60	48
120	52
180	52
**$[C_1C_4SO_3HIm][Cl]$**	
15	47
30	42
60	68
120	57
180	55

[a] Yields are reproducible within ± 4%.

Table S4. Yields in 5-HMF for the dehydration of inulin at 80 °C, in ChCl:GA in the presence of acidic TSILs.[a]

Time (min)	Yield in 5-HMF (%)
**$[C_{222}C_3SO_3HN][Cl]$**	
15	24
30	30
60	39
120	45
180	55
**$[C_{222}C_4SO_3HN][Cl]$**	
15	40
30	42
60	41
120	54
180	52
**$[C_1C_3SO_3HIm][Cl]$**	
15	42
30	39
60	44
120	45
180	55
**$[C_1C_4SO_3HIm][Cl]$**	
15	24
30	38
60	49
120	45
180	51

[a] Yields are reproducible within ± 4%.

Table S5. Yields in 5-HMF for the dehydration of inulin at 80 °C, in ChCl:MA in the presence of acidic TSILs.[a]

Time (min)	Yield in 5-HMF (%)
**$[C_{222}C_3SO_3HN][Cl]$**	
15	43
30	48
60	56
120	53
180	60
**$[C_{222}C_4SO_3HN][Cl]$**	
15	45
30	49
60	49
120	58
180	61
**$[C_1C_3SO_3HIm][Cl]$**	
15	32
30	32
60	43
120	60
180	50
**$[C_1C_4SO_3HIm][Cl]$**	
15	36
30	34
60	50
120	56
180	55

[a] Yields are reproducible within ± 4%.
